# An ontology-driven knowledge graph for tourism information management

**DOI:** 10.12688/openreseurope.17614.2

**Published:** 2025-07-23

**Authors:** SUBHASHIS DAS, Mayukh Bagchi

**Affiliations:** 1 Department of Computer Science and Automation, Universidad de Salamanca, Salamanca, Castile and León, 37007, Spain; 2 Department of Information Engineering and Computer Science (DISI), Universita degli Studi di Trento, Trento, Trentino-Alto Adige/South Tyrol, 38123, Italy

**Keywords:** Tourism, Ontology Modelling, Faceted Ontology, Knowledge Graph, Conceptual Disentanglement, Tourism and Artificial intelligence (AI)

## Abstract

**Background:**

*Lao Tzu*, the ancient Chinese philosopher, had famously said, *“a journey of a thousand miles begins with a single step”*. The modern ecosystem of tourism is *extremely* multi-faceted and multi-context dependent, so much so that tourists, planning on an excursion, have an entire range of queries from as basic as *“Where to eat?”* to as complex as *“List all the eating establishments which are suitable for groups and also provide children menu”*. Such a list becomes even more complicated due to the intermixing of different perceptions, languages, cultures and economies in which tourists are enmeshed in. Accordingly, such increasing complexity in questions from tourists necessitate the need to provide specialized and fine-grained data and information services which are beyond the Information and communications technology (ICT) infrastructures in vogue in tourism today.

**Methods:**

In this paper, we propose and advocate the use of, via a theoretically and implementationally *well-founded methodology*, the Artificial Intelligence (AI) backed technology of *ontology-driven Knowledge Graphs*, to provide fine-grained information services to tourists at any scale and at any level of complexity.

**Results and Conclusions:**

The methodology is validated by developing a fully populated tourism-specific ontology-driven knowledge graph having 4264 RDF statements and ready for *reuse*, *extension* and *exploitation* as a part of any of the current ICT infrastructure in tourism sector.

## Plain Language Summary

Lao Tzu once famously said, “a journey of a thousand miles begins with a single step.” This quote captures the essence of embarking on any journey, including travel. Nowadays, the tourism industry is incredibly complex. When people plan trips, they have a wide range of questions, from simple ones like "Where should I eat?" to complex ones like "Which restaurants are good for groups and offer menus for children?" This complexity is further compounded by the diversity of perceptions, languages, cultures, and economies that tourists encounter.

As a result, there's a growing need for specialized and detailed information services to cater to tourists' varied queries. The current technology used in tourism, like Information and Communication Technology (ICT) infrastructures, may not fully meet these evolving needs.

In this paper, the authors propose using Artificial Intelligence (AI) technology, specifically ontology-driven Knowledge Graphs, to provide tailored information services to tourists regardless of the complexity of their questions. Ontology-driven Knowledge Graphs leverage AI to organize and connect information in a meaningful way, allowing for more nuanced and personalized responses to tourists' inquiries.

The authors validate their proposal by developing a tourism-specific Knowledge Graph populated with over 4000 statements. This Knowledge Graph is designed to be adaptable and expandable, making it a valuable resource for enhancing existing ICT infrastructure within the tourism sector.

In essence, the paper suggests that leveraging AI-driven technology like Knowledge Graphs can revolutionize how tourists access information, providing them with more precise and comprehensive assistance during their travels.

## 1 Introduction

Tourism has facilitated, since time immemorial, both intracultural and intercultural dialogue and exchange amongst otherwise diverse geographies and cultures. Even in its most modern form, tourism has been, and increasingly so, a *“key driver of the global economic recovery, and a vital contributor to job creation, poverty alleviation, environmental protection and multicultural peace and understanding across the globe”* ^1^. An essential desideratum which underlies all of the aforementioned ramifications of tourism involves the availability and exploitation of multi-faceted and multi-contextual *information* [see, for instance, 1, 2]. In fact, it wouldn’t be an overstatement to label *semantically* annotated and structurally *interrelated* information as the *gamechanger* for the success of a touristic excursion, all the more so in the face of *tourism big data* [see 3].

In continuation of the above context, let us consider the motivating example, for instance, of an *information services chatbot* ^4^ specialized in providing information about the touristic *Points of Interest* (PoIs) of a region. We are particularly interested in the *design of the knowledge base (KB)* ^5^ which underpin the multi-faceted informative replies provided by such a chatbot (or, any similar information service-oriented application). There are at least four *pivotal* considerations impacting the design of any such KB. Firstly, there should be a baseline set of *queries* which can be posed to the application (such as, the chatbot) and to which it is expected to factually reply. Secondly, grounded in the above queries, there should be a baseline set of linguistically named *concepts* which compose such a KB and which the KB exploits to generate factual replies. Thirdly, such concepts should be interrelated amongst themselves and organized into a baseline *graph-like conceptual hierarchy* in order to tackle, especially, the more complex queries posed, for example, to the chatbot. Fourthly, and last, each concept in the hierarchy should be described via a baseline set of *attributes* which are exploited to provide *fine-grained* informative answers to the queries vis-`a-vis *ground truth*. Notice that, as a key requirement, each of the above considerations should be *methodologically well-founded* in order to be modifiable and/or evolvable later on (for instance, due to a change in information *sources* or *services* ^6^).

We posit that the fundamental bottleneck afflicting the aforementioned design considerations (individually and in unison) can be mapped to the more fundamental phenomenon of *conceptual entanglement* ^7, 8^ ubiquitous in (semantic) data and information representation(s). Firstly, for any *domain of discourse* ^9^, there is always an unavoidable *manifoldness* (i.e., multiplicity, many-to-many correspondences) between the domain *as such* and its *perception* by an agent (thereby generating *diverse* queries in the process). Secondly, given perception, there is always an unavoidable *manifoldness* between the perceived domain and the linguistically encoded *body of concepts* an agent utilizes to describe it. Thirdly, given the body of concepts, there is always an unavoidable *manifoldness* in how they can be *hierarchically organized and interrelated*. Finally, given the conceptual hierarchy, there is always an unavoidable *manifoldness* in the way in which each individual concept in the hierarchy can be *intensionally characterized* (i.e., via attributes).

Our proposed solution takes the form of an interdisciplinary, step-by-step *faceted ontology modelling methodology* towards *disentanglement* of the conceptual entanglement issues as described and exemplified above. A domain ontology, defined as *“a formal, explicit specification of a shared conceptualization”* ^10^, is essentially a computationally exploitable graph of concepts encoding knowledge about a target domain of discourse at different levels of abstraction. The methodology is founded in the interdisciplinary intersection of theories and technologies from faceted knowledge organization (KO) ^11^, Artificial Intelligence (AI)-based applied ontology ^12^ and semantic web standards such as RDF, OWL and SPARQL ^13^. The key observation is that the methodology, via adapting the best practices individually from tried-and-tested arenas of information and knowledge (base) organization, generates a high-quality ontological knowledge base which is *interoperable* and *reusable* within the larger gamut of *linked open data* ^14^. We also validate our methodology by developing a reusable and expandable *Tourism Applications Ontology (TAO)* which models and integrates real-life tourism data of tourist-specific eating establishments from the Italian province of Trentino into a *Tourism Domain Knowledge Graph (TDKG)* (Knowledge Graphs (KGs) being ontologies populated with data; see 15). The ontology-driven knowledge graph can then be exploited both by *humans*, for gaining structured information specific to different information queries, and by computer *applications* as a high-quality, expandable knowledge base for providing advanced information services. Notice that ontology-driven KGs have founded wide success in tackling socio-technical challenges at different levels of complexity in diverse domains as detailed in 15.

The novelty of the proposed solution can be noted from two different perspectives. Firstly, notice that our methodology being crucially based on the principles of *faceted classification* ^11^ - the principled analysis of a domain of discourse by hierarchically elucidating the *generic terms used to denote its conceptual components* at the requisite level of abstraction - is *innately* capable of disentangling the conceptual entanglement inherent in ontological representations produced by existing state-of-the-art methodologies. Hence, the TAO (at the conceptual level) and the TDKG (at the structured data level) produced following the methodology are taxonomically well-founded and ready to be exploited, expanded and *reused*. The second novelty is the fact that, from a socio-technical perspective, the methodology can provide the starting basis on which an all-encompassing FAIR information organization ecosystem ^16, 17^ can be built which can subsequently be exploited by all players in tourism - tourists, businesses, governments, human and artificial agents, researchers, analysts, etc.

The rest of the paper is organized as follows: Section (2) details the research background of the work founded on a comprehensive review of relevant scientific literature in tourism and Information & Communication Technology (ICT). Section (3) elucidates the proposed faceted ontology modelling methodology in a step-by-step fashion and additionally illustrates each step with running examples grounded in real-world tourism data. Section (4) and Section (5), respectively, provide (via snapshots and illustrations) a glimpse of the technical implementation of the ontology and ontology-driven KG model and an illustrative evaluation of its performance as against (user) queries. Section (6) summarizes and concludes the paper by stressing the socio-technical ramifications of the proposed solution on the tourism domain (especially what concerns its information services).

## 2 Literature review

We organize the literature review around four dimensions. Firstly, we concentrate on works that deal broadly with the applications of ICT and Artificial Intelligence (AI) techniques in tourism information management. Secondly, we focus on literature which have employed some form of ontology and KG model to manage, exploit and analyze tourism data and information. Thirdly, we also review some of the prominent as well as newly proposed ontology and KG development methodologies (in general but also focused, if existent, on tourism). Finally, we also briefly explore the research in KO and its intersection with ontologies and KGs to contextualize and position the *interdisciplinarity* of the current work.

### 2.1 Tourism and ICT

The importance of Information and communications technology (ICT), consensually understood as the computing facilities employed for data(base), information and knowledge(base) management ^18^, in facilitating genuine information access to diverse players in tourism and in allied sectors was stressed in a detailed fashion in 19, 20. The advantages that ICT applications and networked data analysis brings towards the fulfilment of Sustainable Development Goals (SDGs) vis-`a-vis tourism was highlighted in 21. The work in 22 is very interesting from a quantitative perspective as it studied and concluded that ICT infrastructure had a *“positive, statistically significant relationship with tourism development”*, especially, in as large a continent as Africa. The descriptive study in 23 concluded that the tourism industry is heavily influenced by AI technologies, especially by information services *chat-bots* and internet of things (IoT) systems. The recent systematic study of the emerging applications of AI technologies in the tourism sector ^24^ also vindicated the fact that, even though human touch is still the *“determinant of experiential tourism”*, AI technologies are steadily emerging as high-quality complementary support for the future of tourism. The recent study in 25 also explicated the importance of the availability of structured data and information to develop and strengthen AI technologies in the tourism sector.

### 2.2 Tourism and ontology-driven KGs

We now briefly review research works which proposed some form of ontology model to facilitate tourism information management. The Harmonise ontology by 26 was mainly proposed for tourism data exchange. The authors in 27 proposed the QALL-ME technique which used controlled vocabulary and top level ontology in addition to WordNet and SUMO to develop the knowledge base for their information management framework (which can be exploited for any domain including tourism). The use of ontology to find semantic similarity between concepts for use in e-Government tourism service recommendation system has been discussed by 28. 29 listed the existing ontologies on the tourism domain. The Mondeca ontology used concepts from the World Tourism Organization thesaurus. The different concepts related to tourism information dissemination are present in the OnTour ontology, which was developed by the eTourism Semantic Web Portal. An ontology-driven tourism recommender system was designed by 30 where algorithms have been used to deal with context of users. An ontology for intelligent transportation systems was developed by 31 using semantic clustering algorithms. Ontology has been also used for medical and leisure tourism recommender systems as is evident from the works in 32 and 33 respectively. 34 tried to model the context in tourism. More recently, the work in 35 proposed an Event-centric Tourism Knowledge Graph (ETKG) which has the capability to model both the temporal and spatial dynamics of tourist trips.

### 2.3 Ontology and KG development methodologies

Finally, let us concentrate briefly on ontology and KG development methodologies. The survey in 36 provides an overview of some prominent early-generation ontology construction methodologies. The architecture in METHONTOLOGY ^37^ proposed a *“life cycle to build ontologies based in evolving prototypes”*. Ontology Development 101 ^38^, instead, proposed a flexible methodology which allowed to choose and combine top-down, bottom-up or middle-out approaches in modelling ontologies. The NeOn methodology ^39^ offers a set of general use patterns for reuse, remixing and merging of ontological resources. More recently, a modular modular ontology modelling approach was proposed in 40 encouraging the reusability of ontologies. Distinct from the above, the use of facet analysis as the key methodological foundation in mainstream ontology engineering was proposed in 41– 44 and expanded to ontology-driven KG modelling in 45– 47 respectively. Finally, a recent paper ^48^, though in a slightly different yet related context, elucidated the key critical advantages and differences amongst (some of) the different methodologies referenced above. The present work also exploits the longstanding body of research on foundational ontologies (see, 49– 51) and exploits, in particular, the proposal advanced by 49. Two observations. First, notice that while none of the above methodologies or ontological theories were instantiated to tourism data/information, all of them can be, potentially, with requisite tweaks, applied to model and analyze tourism data. Second, none of the above research considers the full spectrum of conceptual entanglement from perception to intensionality (possibly, due to their different goals or focus) and possible solution strategies to disentangle existing entanglement.

### 2.4 KO and ontology-driven KG development

KO, defined as *“description, indexing and classification performed in libraries, bibliographical databases”* ^52^, has long been a crucial component of any information management system (whether manual or (semi)automatic). In addition to the concrete classification and indexing systems KO has developed (see, e.g., 53– 55) there has been a lot of foundational research on domains, facet analysis and how they can be exploited as a crucial part of any well-founded methodological pipeline to manage data and information. For instance, the very notion of domain analysis ^56^ and its extended application to modern ontology-based knowledge organization formalisms ^57^, is functionally linked to *perception* as discussed in conceptual entanglement and can be potentially adapted (in future works) to provide a foundation to disentangle perceptual manifoldness. Within the various theories of knowledge organization (see 58 for an overview), the facet-analytical paradigm of organizing and representing knowledge originally advanced by Ranganathan ^59, 60^ is key to how, in our methodology, the ontological hierarchy is modelled via facets and specialization of (sub)facets, thereby, facilitating disentanglement of manifoldness in concepts and ontological hierarchies. In fact, recent cumulative work in knowledge organization on how perception, language and ontological hierarchies are functionally linked (see 61– 65) has also been key to understand our proposed facet-based ontology modelling methodology. Finally, our work is also orthogonal to recent research within KO as to how the foundational theories and methodologies of KO can be leveraged to model semantically well-founded and ontologically explainable KGs (see, for instance, 66– 68). Notice, however, the fact that from none of the above research has been directly instantiated to tourism-based data and information. To that end, the present work is a first preliminary effort.

Chessa *et al.* (2023) ^69^ propose a robust data-driven methodology for automatically generating tourism-specific knowledge graphs. Their approach leverages structured and unstructured data sources and integrates schema design, data extraction, and KG population into a single pipeline. This work exemplifies how existing methodologies can be pragmatically adapted and implemented in tourism scenarios without necessarily reengineering the conceptual modeling process. Simsek *et al*. (2022) ^70^ offer a broad knowledge graph engineering perspective, including insights into domain-specific applications such as tourism. Their work emphasizes practical design patterns, toolchains, and knowledge reuse strategies, showing that existing frameworks can be effectively instantiated even in complex domains like tourism without extensive foundational modifications.

### 2.5 Critical analysis of research gaps

We noticed three research gaps within the aforementioned literature that we reviewed. Firstly, from the broad perspective of ICT and AI applications in tourism data and information management, there is a major *under-utilization* of semantic technologies and semantic knowledge management methodologies. This has consequential (mostly, negative) effects on annotating, modelling, using, analyzing, and reusing tourism data and information by almost all players party to the tourism sector. Secondly, even when some form of ontology (aka KG schema) formalization (or, semantic technologies in general) is employed to model tourism data/information, there is no *methodological framework* to harmonize different perspectives of the same target reality of tourism, resulting in a *conceptually entangled* representation suitable for some and unsuitable for the rest. Thirdly, none of the methodologies surveyed above provides an explicit, step-by-step approach to disentangle the conceptually entangled ontology (aka KG schema) models that are produced (this, being a major factor behind the lack of reusability and expandability of such models).

### 2.6 Novelty

Notice that, as per the aforementioned overviews of the different dimensions in research literature, the applications of ontologies, conceptual models and/or KGs for tourism data and information modelling are *limited*, and, when some form of ontological model is employed, the focus is on the concrete knowledge artifact (i.e., a specific ontological model or KG) *and not* on the underlying general methodological approach to support its implementation and potential reuse. In fact, none of the above research papers encompass within a single methodological framework the crucial dimensions of *conceptual disentanglement* as follows:

1. *Perception*, a key level towards eliciting the precise purpose behind the ontology-driven knowledge base modelling exercise, thereby, reducing perceptual ambiguity;

2. *Concepts*, a key (knowledge) representational level to elicit how the perception and analysis of a domain (e.g., tourism) translates into semantically unambiguous concepts (classes and properties), thereby, reducing terminological ambiguity;

3. *Hierarchy* which, assuming a shared perception and body of concepts, is important to determine both the *ontological commitment* of concepts ^49^ (i.e., whether a concept is an artifact, event, mental object, etc.) as well as the precise hierarchical organization and subsumption of the concepts, thereby, eliminating ontological and hierarchical ambiguity, and,

4. *Intensionality* which, assuming all the above levels, is crucial to model the object properties and data properties which interrelated and describe each concept in the hierarchy, thereby, eliminating intensional ambiguity.

The current work is a first attempt to advance a conceptual approach and a well-founded and exemplified methodology to tackle the complexity of the aforementioned levels of conceptual (dis)entanglement within a single approach within the spectrum of tourism data and information. To that end, the first step of the methodology, namely, domain and competency question identification explicates the otherwise implicit and entangled perception of the domain (here, tourism) specific to the purpose of the design of the ontological model composing a knowledge base. The second step focuses on precisely explicating the otherwise implicit and entangled (linguistically labelled) body concepts which would constitute the ontology-based tourism KG. The third step of the methodology concentrates on explicating the otherwise implicit and entangled ontological and taxonomical assumptions which complicate and potentially entangled the development of an ontology-driven tourism KG. The subsequent steps of the methodology focuses on using standard KR and ontology modelling technologies to model an instantiation of the concrete tourism ontology and ontology-driven KG. Let us now concentrate on the concrete methodology.

## 3 Methods

As a unified, *well-founded solution* to the aforementioned research gaps, we now concentrate on the steps of our proposed faceted ontology modelling methodology. Before elucidating and illustrating each step, we ground the applicability and exploitability of the methodology in a concrete real-life research background, namely, the different genres of eating establishments available for tourists to choose from while visiting the Italian province of Trentino.

### 3.1 Research background

The proposed methodology and the base version of the ontology-driven KG model developed following the methodology are mainly inspired from datasets ^2^ on tourist-specific eating establishments situated in the Italian province of Trentino Alto-Adige ^3^ (such as, from the data catalog OpenData Trentino). The conceptual analysis of such datasets resulted in a first set of concepts related to tourist-specific *eating establishments* as well as bootstrapping a first version of the hierarchical taxonomy to be adopted as the backbone of the ontology/KG model. However, a central lacking of the datasets was that they contained only very basic information (such as, for instance, the address and the telephone number) about the tourist-specific eating establishments. Therefore, we decided to scrape other sources such as Tripadvisor and Yelp in order to extend the informational attributes provided by OpenData Trentino (and consequently, to design a more generalized ontology i.e. KG schema model). The names of the eating establishments and their geographical placement, including city and address, were freely available on OpenStreetMap and therefore, no copyright infringement occurred. However, some of the labels of the additionally incorporated attributes and fields were altered to avoid copyright infringement. Moreover, synthetic data was created when no open information were found. In addition to the above, further inspiration came from the schema.org concepts of *Food Establishment* ^4^ and from the *BBC ontology on Food* ^5^. These, along with the previously cited sources of inspiration, were reused and appropriately incorporated as reference information modelling standards. The stress on reusing such standards, in particular, was in order to make data integration, i.e. Integrating data from multiple sources to provide users with a unified view ^71^, as efficient as possible. Given the aforementioned research background, we now sequentially elucidate and illustrate each step of our proposed methodology.

### 3.2 Step-1: Domain and competency question identification

This step initiates the proposed methodology by conducting a detailed analysis of the domain of interest ^72^ of which the data is to be structured and modelled as a KB. Such an analysis, in addition to a preliminary conceptual clarification regarding the types of concepts, relations and attributes to be included, also yields concrete requirements modelled as *Competency Questions* (CQs) ^73^. A CQ is thus encoded as a natural language sentence that defines a pattern for the types of questions users anticipate an ontology-driven knowledge graph-based knowledge base to address.

For example, tourism as a domain is very vast and therefore it is vital to identify *CQs* as concrete requirements in order to define the boundaries of tourism that one wants to conceptually model as an ontology-driven KG. Consequently, the queries that a user might want to perform on such an ontology-driven KG (such as, particular to tourist-specific eating establishments) are also many and can be of different nature. We mention some illustrative CQs which were found to be interesting as well as simple to elucidate the methodology.

A general CQ, may be of more interest to foreign tourists, could be *to have a list of the types of eating facilities present in Trento*. This is especially interesting as different countries might have different types of eating establishments (some of which might not exist elsewhere). A prominent example is represented by *trattorias* and *osterias* which are characteristic of Italy. Another CQ that one might want to model in the ontology-driven KG is to *find all places where one can eat a certain meal (e.g. breakfast, lunch or dinner) in Trento or in one of the Trento’s suburbs*. A specific example of this type of query is *“Where is it possible to eat lunch in Cognola?”*. It is also possible to ask even more specific queries, such as “Where is it possible to eat pizza for lunch in Cognola?”. Notice that, as already alluded to in the introduction, this step is crucial to disentangle the entanglement evident in the perception of a domain (such as tourism).

In fact, queries can be performed to elicit information from the ontology-driven *TDKG* about ratings, prices and amenities of the eating establishments at different levels of abstraction. Some illustrative examples of such queries are listed as follows:

• *CQ1:* “Whether a transportation stop has wheelchair access?”

• *CQ2:* “Whether bicycle is allowed in the trip?”

• *CQ3:* “List all the eating establishments which are suitable for groups and also provide children menu.”

• *CQ4:* “What are the nearest eating establishments surrounding a trans-portation stop?”

• *CQ5:* “List all the places where one can eat which have ratings higher than 4 stars.”

• *CQ6:* “Give me eating establishments with vegetarian menu and price range medium-low.”

• *CQ7:* “Give me the contacts of all the eating establishments that accept reservations and have a parking lot.”

As seen from above, these queries not only provide very useful information about eating establishments, but as in the latter examples, they may even provide the direct means to contact the eating establishment. Further, in order to fulfill user expectations, queries might provide options for specifying filters such as menu type or suitability for a specific occasion, such as a romantic date or a business meeting. There can be further queries that can involve the events that are held in eating establishments. For example, one can ask *“Which events will be scheduled in this eating establishment in the future?”*. Or, similarly, given the knowledge of an event, one might ask *“Where and when will this event be held?”*. Tourists might also be interested in knowing whether some famous or extraordinary events were held in the place they are eating right at the moment.

The list of CQs presented above, although already complex to a certain degree, might get even more fine-grained if the domain is expanded in order to include other, for instance, complementary domains. Examples of possible expansion are the domain of transportation, which might be exploited in order to allow the user to know how to reach a certain eating establishment, or the domain of food ^74^. Notice also the fact that these CQs perfectly map to our introductory motivating example of developing an ontological KB for a tourism information services chatbot that can provide informative answers to user queries. Finally, observe how this step of domain analysis and competency question identification is functionally linked to explicit disentanglement of the otherwise implicit entanglement and manifoldness observed in instantiations of perception (e.g., in the form queries) in mainstream ontological models and KGs on tourism.

### 3.3 Step-2: Concept vocabulary determination

Given the conceptual analysis of the domain (e.g., tourism) and the elicitation of requirements as CQs, the second step of the methodology - *concept vocabulary determination* - is focused on choosing *standardized linguistic labels* with unambiguous meaning from relevant lexical resources ^75^ and/or controlled vocabularies ^76^ for encoding the concepts expressed in natural language CQs. This facilitates disentanglement of the entanglement evident in the linguistic labelling of the perception of a domain (such as, tourism).

Given the elicited CQs, the main source of the concept vocabulary used for the definition of the terminology used in order to build the *TAO* and later *TDKG* was WordNet ^77^, a large lexical database of English where nouns ^6^ are organized into sets of cognitive synonyms (synsets), each representing a unique concept. An exception was made for *osteria* and *trattoria*, two forms of eating establishments typical of Italy. These words, belonging to the Italian language, are also adopted for use in English, as a proper translation is not possible due to the well-known linguistic phenomenon of *lexical gaps* ^78^. Since WordNet and other sources, such as Oxford Dictionary, provided only a generic definition of *osteria* and *trattoria* as an *“an Italian restaurant”*, it was decided to use Wikipedia’s definition for these two eating establishments as it was the only source providing a clear meaning on what these two concepts are.

The standardized disentanglement of the semantics of linguistic labels encoding perceived concepts is key to mitigate the *semantic heterogeneity* problem, i.e., the difficulty of establishing a certain level of connectivity between people, agents or software systems ^79^ for the purpose of enabling each of the parties to the appropriately understand exchanged information ^80^, e.g., as in tourism domain.

### 3.4 Step-3: Faceted hierarchy creation

**Figure 1.**
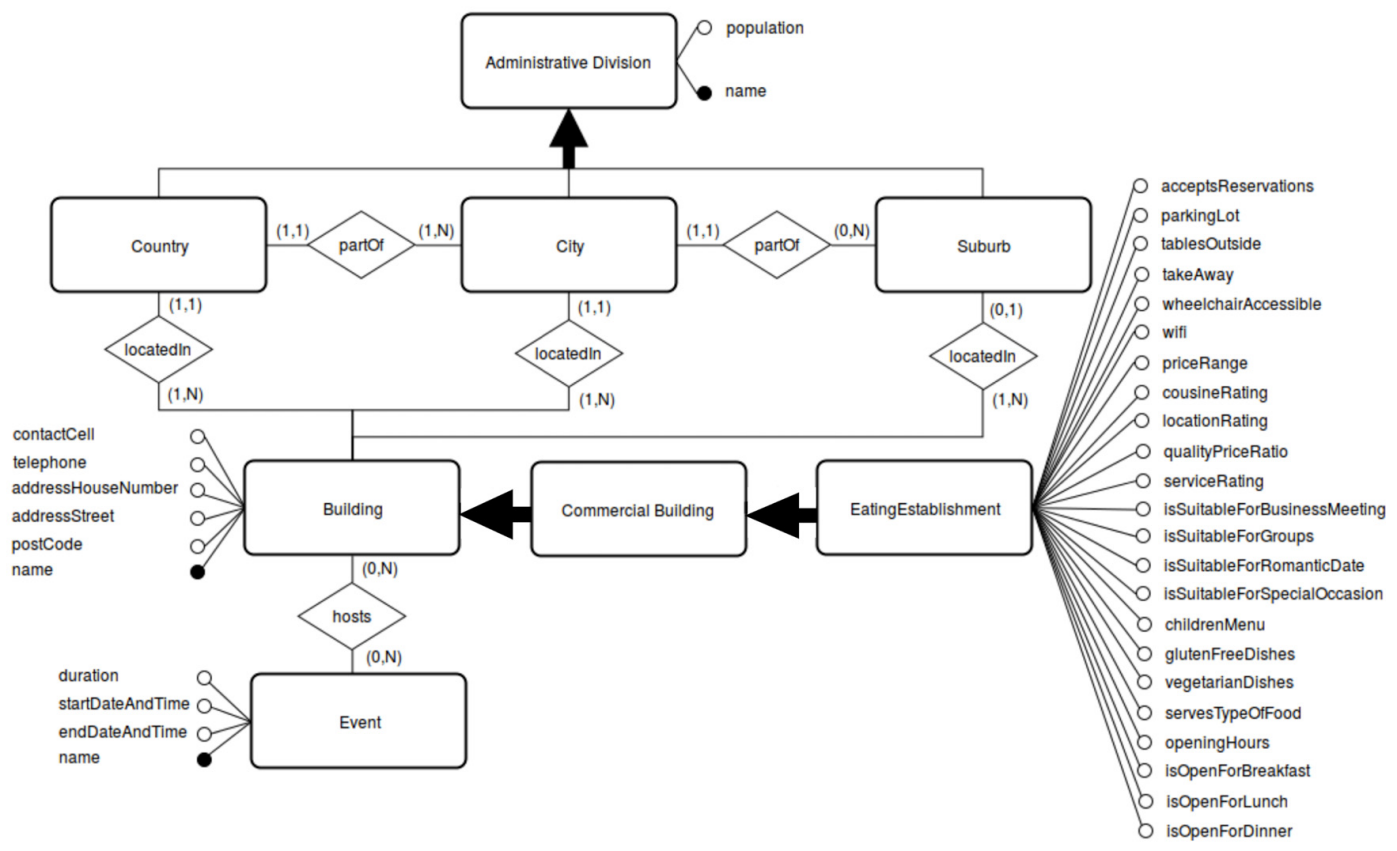
A partial illustration of the Extended ER (EER) Model.

Given the elucidation of CQs coupled with the aforementioned concept vocabulary creation, the *faceted hierarchy creation* step models the interrelated hierarchy of classes, relations, and attributes from amongst the unambiguous concept labels obtained in Step (2). The methodology followed to model the classes, relations, and attributes is an adaptation of *faceted classification* ^11^, a *tried-and-tested* dynamic knowledge organization methodological framework used to classify and interrelate information resources in libraries and information institutions. The illustration of the conceptual hierarchy obtained as a result is illustrated in Figure 1. Notice that this step of facet creation is extremely crucial to disentangle the entanglement implicit in the hierarchical model and intensional definition of any ontology-driven KG-based KB. We now provide an explanation for each of the three sub-components of facet creation, namely, class, attributes, and relations.

***3.4.1 Class.*** Firstly, we model *classes* which represent *“concepts in the domain”* ^38^. Some of the classes we considered for *TAO* and which were exploited to model *TDKG* are illustrated as follows:

• *Eating Establishment:* This class describes a general place where people go to eat. It is a specialization of *commercial building* which in turn is a specialization of a *building*. They can be further specialized into several (sub-)classes depending on the differentiating characteristics of the eatery itself, which, in some cases can be very subtle. A list of such sub-classes are presented below:

    – *Bar:* A bar is defined as either a counter where food or drinks can be obtained or a space or establishment where alcoholic beverages are served over a counter.

    – *Bistro:* Bistros are small and informal restaurants that typically serve wine.

    – *Cafe:* A Cafe is similar to a bar but additionally house a small restaurant where drinks and snacks are sold.

    – *Fast Food Joint:* Fast food joints are eating establishments where inexpensive food, such as hamburgers or chicken or milkshakes, are prepared and served quickly.

    – *Alpine Hut:* Alpine huts are characterized by their geographical position as they are usually located high up in the mountains and are usually accessible only by foot. They are intended to provide food and shelter to tourists who intend to engage in mountaineering, climbing and/or hiking.

         Notice also that this concept is more *situational* given that

    – *Osteria:* Osterias, as noted beforehand, are typical of Italy. It is sometimes roughly translated to be an inn or tavern. Osterias were originally establishments offering wine and simple meals. Recently, the emphasis has shifted to food, with menus typically remaining concise and highlighting local specialties like pasta, grilled meat, or fish, often served at communal tables. *Pizzeria:* Pizzerias are also originally from Italy but are now commonplace in the rest of the world. They are shops where pizzas are made and sold.

    – *Pub:* This type of eating establishment is comparable to a tavern which comprises a building with a bar and public rooms. In pubs, light meals are often provided.

    – *Steakhouse:* Steakhouses or chophouses generally serve exclusively meat dishes, typically steaks or chops.

    – *Trattoria:* Trattoria is defined as an Italian-style eating establishment, less formal than a restaurant, but more formal than an osteria. Generally, there are no printed menus and wine is sold by the decanter rather than the bottle. Typically, the service is casual and prices are low with the food served being plentiful, mostly following regional and local recipes. In some instances, trattorias even serve food in family-style, i.e., at common tables.

    – *Restaurant:* This is a quite general type of eating establishment and is simply defined as a building where people go to eat. Although the definition is *similar* as the one of an eating establishment, it was decided to keep it separate from the main class as in the specific context of Italian culture, it can represent a unified class which can be a (multiple) combination of any of the above classes.

• *Event:* An event is something that happens at a given place and time. We have reused the notion of an *event* as described in the top-level ontology *DOLCE* ^81^. An event can be further divided into several sub-classes depending either on the theme or the people it is devoted to. For instance, a children’s event is an event devoted to children, whereas, a cultural event relates to the arts and aesthetics that a community practices. A seminar is a special type of cultural event that has the goal of exchanging ideas on a specific theme. A social event, instead, is an event attended by sociable people and has the purpose of sociability. Examples of themes that might characterize an event are music or food.

• *Administrative Division:* Administrative or territorial division is an entity that contains all sets of districts defined for administrative purposes. Examples of these districts used in *TAO*-driven *TDKG* are Country (e.g., Italy), City (e.g., Trento) and Suburb (e.g., Povo, Villazzano and Cognola).

***3.4.2 Attributes (Data properties).*** Attributes (aka data properties) are associated with classes in order to assert their distinctive characteristics. In the proposed *TAO*-driven *TDKG*, the classes for which attributes were defined are *Administrative Division, Building, Commercial Building, Eating Establishment* and *Event*. These attributes can be seen in Figure 1 and are listed as follows:

•   We modelled several amenity-specific attributes which include any feature that provides comfort, convenience, or pleasure to tourists. Among these are whether an establishment has a parking lot, tables outside, a takeaway service and/or free WiFi. Further, we modelled attributes which also encoded information as to whether an eating establishment is wheelchair accessible and whether it accepts reservation.

•   We also modelled attributes that allows users to retrieve information on how to contact the selected eating establishment via a cell phone or telephone number.

•   The *is suitable for* attributes was used in order to group all the possible occasions for which an eating establishment might or might not be suitable. Such occasions include business meetings, romantic dates and/or special occasions (each of which are crucial for different genres of tourism ^82^). The latter is typically used for tourist-specific celebratory gatherings and can also be used to encode whether an eating establishment is suitable for numerous groups.

•   Several attributes were modelled to encode menu options, including, for instance, the different types of menus that might be available in an eating establishment, e.g. children’s menu, vegetarian dishes, gluten-free dishes. It can also be exploited to encode the type of cuisine that is served, e.g. Italian, American, Thai etc.

•   Rating, as the name suggests, contains numerical evaluations for elements related to the eating establishments such as the cuisine, the service, the location and quality-price ratio.

•   Spatial attributes modelled included data such as the address street, the house number and the post code.

•   Temporal attributes were used for encoding information related to dates and/or time. It includes attributes for both eating establishments and events. For the former, the opening hours attribute was used in order to list when an eating establishment is open to the public. Moreover, three attributes were used to formally specify whether a place is open for breakfast, lunch and dinner, respectively. For an event which tourists might be interested in, duration (i.e., the number of days), start date-time and end date-time were specified.

Additionally, several other attributes, that were modelled in the *TAO*-driven *TDKG* were *population* (used to capture how many people reside in each administrative division) and *price range* (specific to an eating establishment). The latter is crucial, for instance, for a tourist-specific information services chatbot to encode how expensive an eating establishment is, for instance, in terms of ‘Low’, ‘Medium’, ‘High’, ‘Medium-Low’ and ‘Medium-High’.

***3.4.3 Relations (Object properties).*** Relations (aka object properties) are employed to inter-relate classes among each other as inferred from the requirements modelled as CQs. For example, in our proposed *TAO*-driven *TDKG*, the *part-of* relation has been modelled to encode the inter-relation between a Suburb and a City and between a City and a Country. This is used to express, for instance, that Povo is part of Trento, and Trento is part of Italy. Similarly, the relations *addressCountry*, *addressCity*, and *addressSuburb* are employed to specify geographical locations (the country, city and suburb) of a building. As for the events, we assumed that the events we are interested in are all hosted in a building. Therefore, the hosts and *isHostedBy* relations, which are symmetrical, were defined. There were several other relations modelled in the *TAO*-driven *TDKG* which we don’t describe in detail here, such as, *operatedBy*, *stopeAt*, *origin* and *destination*.

Finally, observe how this step of faceted hierarchy creation is functionally linked to explicit disentanglement of the otherwise implicit entanglement and manifoldness observed in two aspects of tourism ontologies and KGs: their ontological commitment and the explainability behind their chosen subsumption hierarchy.

### 3.5 Step-4: Informal modelling

The informal modeling step is a flexible and repeatable phase where the ontology-driven KG-based KB modeller can interact with different representative stakeholders having differing view points of the domain of discourse (e.g., different players in the tourism sector) to *tune*, consolidate and produce a unified disentangled conceptual model. This tuning is performed using the *Extended Entity-Relationship (EER)* model, an extension of the ER model originally proposed by 83. An Extended Entity-Relationship model is a conceptual data model designed to capture the data requirements for a new knowledge base-driven information system using clear and intuitive graphical notation. The result of the tuning and consolidation performed for tourist-sepcific eating establishments can be partially seen in Figure 1. Notice that, oftentimes, this step yields a harmonized conceptual model encoding different mutually complementary conceptual views (via classes, attributes and relations) achieved in achieved in Step (3) and is, thus, crucial for validating the stratified conceptual disentanglement performed in the previous step. A crucial observation. There have been recent proposals (see, e.g., 40, 84) clarifying and advocating the foundations and processes involved in modelling ontologies by reusing existing ontological models or fragments of ontologies. For example, the proposal in 40 advances a step-by-step general workflow to model ontologies by reusing ontology design patterns ^85^ via a combination of project teamwork and specialized ontology engineering implemented by ontology engineers. The current work has similarities and differences with proposals like the one above. First, the methodology proposed by the current work, though instantiated for tourism domain, is generic and scope, and, therefore, has partial commonalities with any other ontology modelling methodology/process/workflow (including the one above). The *key* difference is, however, the diagnosis and acknowledgement of the stratified problem of conceptual entanglement across the functionally interlinked levels of representation (perception, terminology and concepts, hierarchy, intensionality) and its mapping as to how the proposed facet-analysis based methodology can aid in disentangling the entanglement in each level. In fact, based on the stratification of conceptual entanglement, the reusability of *conceptually disentangled* ontologies and ontology-driven KGs would also be equally stratified: resuability at the level of perception and/or terminological concepts and/or facets and faceted hierarchy and/or intensionality and/or any combinations of the above.

Finally, note that while the methodology is presented in a linear step-by-step fashion in the description (for the sake of simplicity), it is flexible enough to be repeated again and again in a cyclic fashion. To that end, the *TAO* and *TDKG* (described in the next section) versions modelled in a first cycle of the methodology can be, for instance, enriched and expanded with more concepts, properties and corresponding entities if there is a need for the KG to target extended coverage of specific tourism data. Similarly, the reverse can be achieved in subsequent cycles of the methodology if there is a requirement for the KG-backed tourism information service to cover a very specialized tourism theme, e.g., within an exclusive tourism zone.

## 4 Implementation of *TAO* and *TDKG*

We now validate our proposed methodology by computationally implementing the informal conceptual model arrived in Step (4) of the proposed methodology described and partially illustrated in the previous section.

The computational implementation of the informal conceptual modelling was effectuated via Prot´eg´e, an open-source ontology editor freely available, developed by the Stanford Center for Biomedical Informatics Research at Stanford University School of Medicine. Prot´eg´e employs the OWL ontology language as its underlying Knowledge Representation (KR) formalism. An OWL ontology is composed of three principle elements: individuals, properties (which are divided into object properties and datatype properties) and classes, thereby, mapping one-to-one with our proposed methodology. Individuals represent the objects within a domain, properties define binary relations between them, and classes are interpreted as sets encompassing these individuals. The naming convention that we adopted for modelling the classes, attributes and relations in the OWL version of *TAO* via the Prot´eg´e GUI was grounded in the notation *camelCase*.

**Figure 2.**
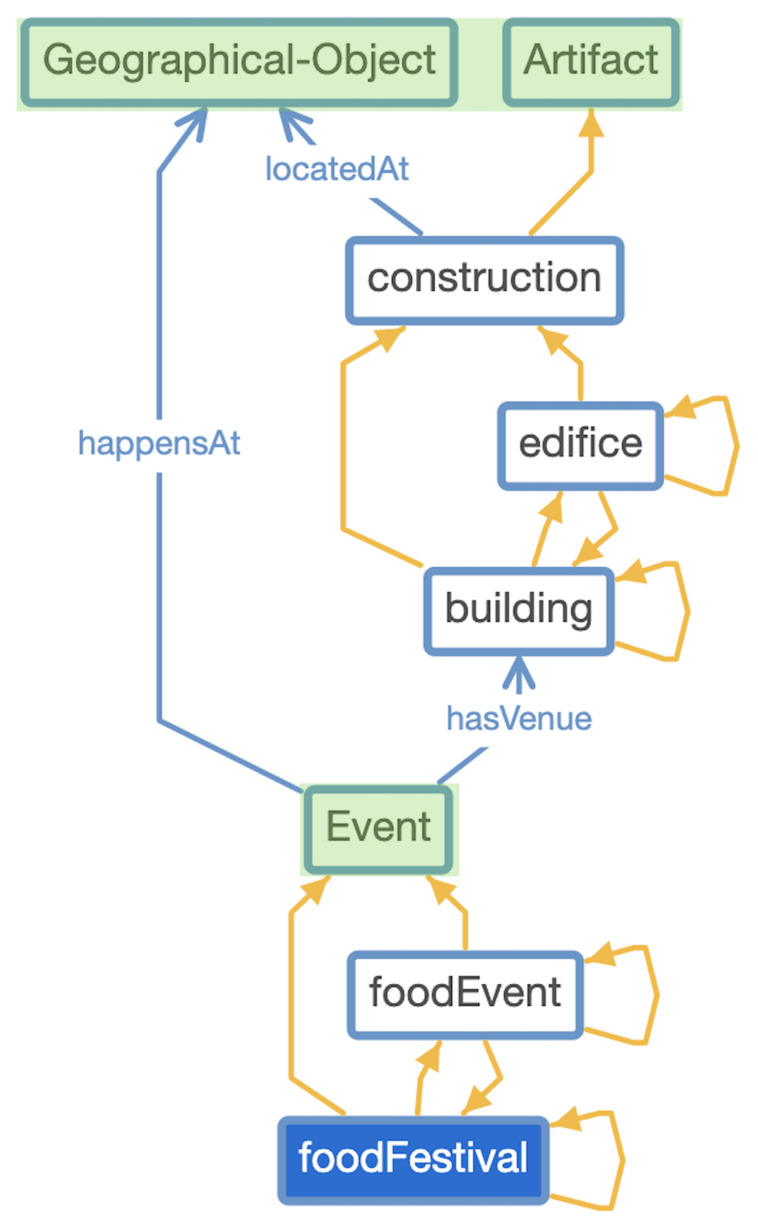
*TAO* alignment with DOLCE Top-level Ontology (DOLCE classes are in snap green).

All the entities described in Section 3.4.1 were modeled as OWL classes in the OWL version of *TAO*. For some of the classes mentioned in Section 3.4.1, we modelled their Prot´eg´e GUI labels after the preferred names of their semantically equivalent synsets as defined in WordNet. This was done in order to allow, for instance, unhindered data integration in the case of the expansion of the datasets which compose the *TAO*-driven *Tourism Domain Knowledge Graph*. In fact, different sources often use different labels for the same concept. For some classes such as artifact, event and geographical object, *logical disjunction* was defined in order to formalize the fact that these classes represent completely disjoint sets (and thereby, completely different concepts in the target reality). Similarly, disjunction was also defined between *country, city* and *suburb*. On the contrary, for sub-classes of events and of eating establishments, it made no sense to define disjunction due to the fact that a single instance might belong to more than one of these sub-classes. For instance, it is common to have a restaurant that is also a pizzeria, and therefore, the instance needs to be modelled in both classes. Moreover, an alignment with DOLCE top-level ontology ^49^ was formalized, as can be seen in Figure 2. This is *key* because of the fact that top-level ontologies describe abstract concepts which not only provides the knowledge model with the desired *formal ontological semantics* but also facilitates, non-trivially, data and information interoperability ^86^.

The relations that were defined in Section 3.4.3 were modelled in the OWL version of *TAO* as object properties. Since it is not desirable to have two relations with the same linguistic label, and since *partOf* is supposed to be used both between city and country and between suburb and city, one of these was modified into *Part of*. While building *TAO*, the cardinalities defined in the EER model in Figure 1 were respected using property restriction constraints as is standard in OWL ontology modelling ^87^. The attributes, that can be seen both in Subsection (3.3) and in Figure 1 with a direct connection to the classes they belong to, were modelled as datatype properties in Prot´eg´e. In specific, all the attributes for the class *building* were defined as strings. For events, instead, the properties *start date-time* and *end date-time* were modeled with a with the *dateTime* data type, while for the duration, which represents the number of days a tourist event can last, integer format was used. The integer data type was also used for encoding the population of the administrative divisions. For most of the attributes of the eating establishments, however, the data type employed was *boolean*. Exceptions are represented by the attributes *servesTypeOfFood* and *openingHours* which were modelled strings. The attribute *priceRange* was also modelled as a string, but, additionally, a restriction was implemented in order to allow the selection of one amongst the following five strings: *Low, Medium-Low, Medium, Medium-High, High*. Similarly, for the attribute *rating* which was modelled as a *float*, restrictions were used to allow only these values: *1, 1.5, 2, 2.5, 3, 3.5, 4, 4.5 and 5*. The OWL version of the *TAO* ontology can be accessed here. TAO ontology has Axiom 1172, Logical axiom count 570, Declaration axioms count 351, Class count 186, Object property count 39 and Data property count 76. The full populated Knowledge Graph has 4264 RDF statements.

**Figure 3.**
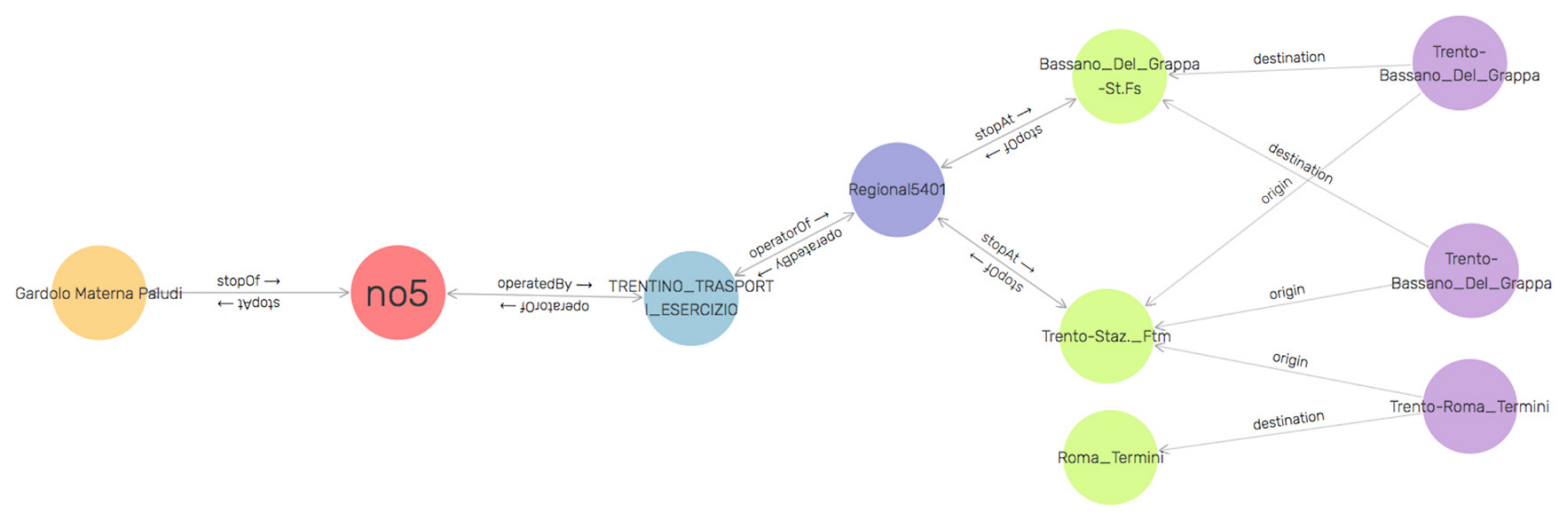
A fragment of the Tourism Domain Knowledge Graph (TDKG) focused on transportation.

Given the mapping and population of the *Tourism Applications Ontology (TAO)* with relevant datasets ^7^, we obtained the *Tourism Domain Knowledge Graph (TDKG)*. The data mapping and population was done semi-automatically using the free and openly-available data mapping tool *Karma* ^88^. A fragment of the *TDKG* specific to eating establishments is illustrated in Figure 3. The GraphDB SPARQL construct has been used to create the visualization of *TDKG*. It is to show that how different elements of the *TDKG* are interrelated at the data level. From the graph, the tourist, for instance, can get information that *bus number 5* stops at *Gardolo Materna Paludi* and this bus service is operated by *Trentino Transporti*. It further shows that *Trentino Transporti* also operates regional train service (e.g. Regional5401) which stops at stations *Trento Station FTM* and *Bassano Del Grappa FS*. Other information that we can from the visualized fragment of the *TDKG* is the name of origin and destination of a train trip (e.g. TrentoRoma Termini). The illustration of the entire *TAO* and *TDKG* was omitted as it results in an extremely complex visualization (also, not the focus of this paper).

Finally, having computationally implemented *TAO* and *TDKG*, we are able to produce SPARQL queries ^89^ ^8^ which are computational query renderings of the natural language CQs elicited in Step (1) of the methodology. The performed queries and the results obtained can be seen in the next section.

## 5 Evaluation

We performed an evaluation of the ontology and the ontology-driven KG both as computational artifacts but also as a *proxy* for the validity of the proposed methodology itself.

For the first aspect, we followed the evaluatory guidelines (both qualitative and quantitative) proposed by Gómez-Pérez ^90^. According to Gómez-Pérez ^90^, the overall goal of evaluating an ontology development process is to check whether the developed ontology definition is, cassificatorily as well as semantically, correct or incomplete or largely incorrect. In concrete terms, the two steps to be followed are: *verification and validation*. The purpose of verification is to check the computational *classificatory correctness* of the developed ontology-driven KG. The purpose of validation, instead, is to check its consistency, completeness, and conciseness. We ascertained the verification dimension of our model via the facility provided by the ontology editor Protégé, by using available reasoners such as HermiT and Fact++ which are available as plugins. The model is concise if it does not accommodate redundancies. In fact, our ontology-driven KG model was found to be largely complete and concise due to careful incorporation of the necessary entity types and properties from the competency questions, thus, capturing what it was purported to represent of the target fragment of tourism. On the other hand, the semantic quality of the ontological commitment (and thereby, of the ontology-driven KG) in terms of consistent modelling of meta-properties like *identity* and *(non)rigidity* was checked and ensured following the *OntoClean* methodology suggested by Guarino and Welty ^91^.

**Figure 4.**
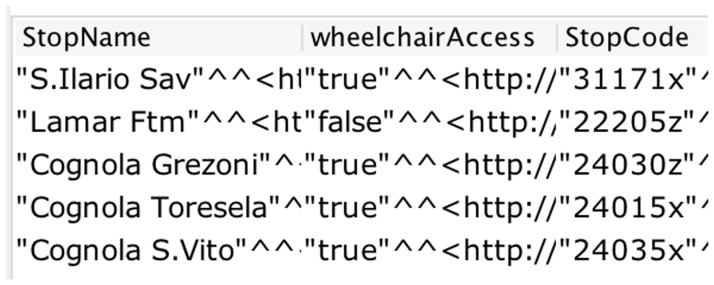
A snapshot of the SPARQL query result for wheelchair access.

For the second aspect, we evaluated our ontology-driven KG by modelling the CQs elicited from Step (1) in Section 3.2 as SPARQL queries ^89^ and adjudicating the answers returned. In fact, to answer *CQ1*, we attached the data property *wheelchairAccess* with the class *transportationPoint* and set the datatype of the data property as Boolean. A snapshot of the SPARQL query structure and the corresponding answer is shown in Figure 4.


PREFIX rdf : *<*http://www.w3.org/rdf−syntax−ns#*>*
PREFIX owl:*<* http://www.w3.org/2002/07/owl#*>*
PREFIX rdfs : *<*http://www.w3.org/2000/01/rdf−schema#*>*
PREFIX xsd:*<* http://www.w3.org/2001/XMLSchema#*>*
PREFIX gtfs : *<* http://purl.org/net/tour−gtfs#*>*
SELECT? StopCode? StopName? wheelchair Access
WHERE *{*
 ? bus Stop gtfs : stopCode? StopCode.
 ? bus Stop gfs : name? StopName.
 ? bus Stop gtfs : wheelchair Access? wheelchair Access.
*}*ORDERBY?name LIMIT 5



**Figure 5.**
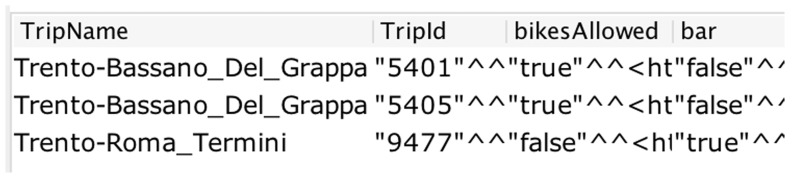
A snapshot of the SPARQL query result for bikes allowed.

Similarly, for *CQ2*, we created *bikesAllowed* as a datatype property and attached it with the class *trip*. Figure 5 shows a snapshot of the result from the query.


PREFIX rdf : *<*http://www.w3.org/rdf *>*
PREFIX owl : *<*http://www.w3.org/2002/07/owl#*>*
PREFIX rdfs : *<*http://www.w3.org/2000/01/rdf−schema#*>*
PREFIX xsd : *<*http://www.w3.org/2001/XMLSchema#*>*
PREFIX gtfs : *<*http://purl.org/net/tour−gtfs#*>*
SELECT? TripName? Trip Id? bikes Allowed? bar
WHERE *{* ? TripName gtfs:trip Id? Trip Id.
       ? TripName gtfs:bikes Allowed? bikes Allowed.
? TripName  gtfs:bar? bar *}*


**Figure A1.**
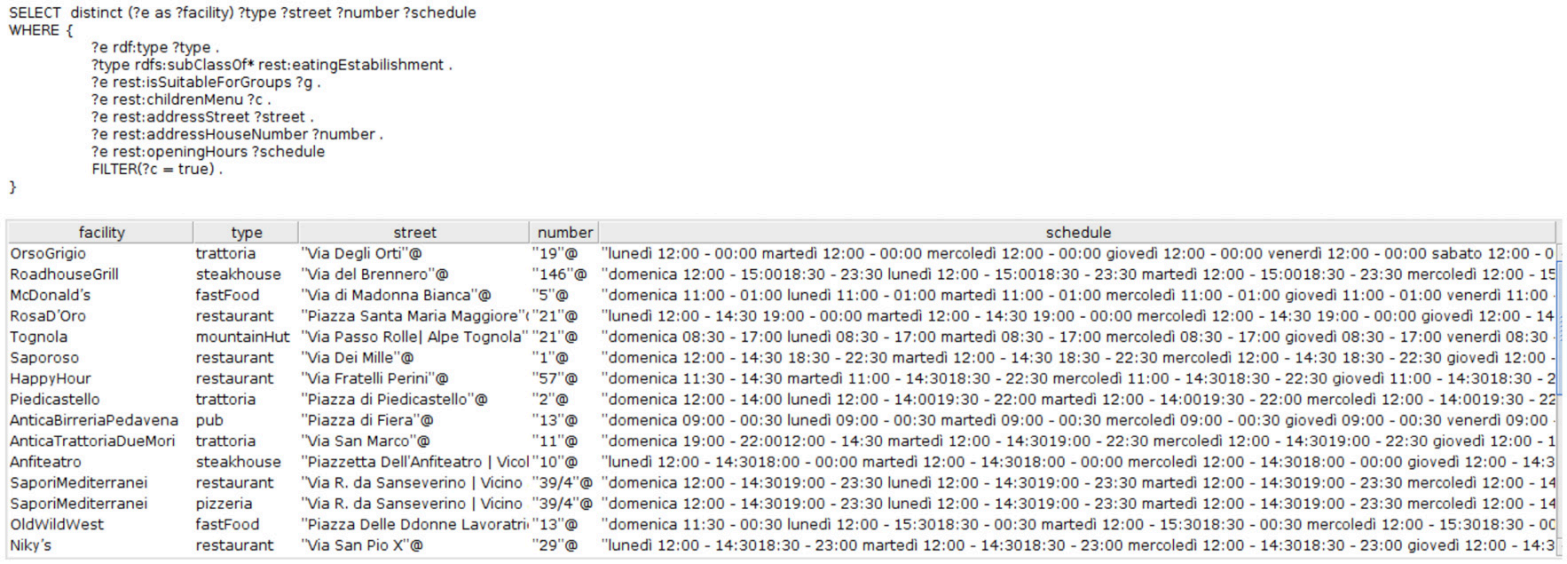
snapshot of the SPARQL query result for eateries suitable for groups and that have children menu.


**Figure 6.**
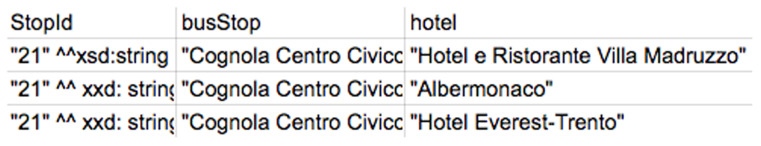
A snapshot of the SPARQL query result for near by services.

The results of the SPARQL query for *CQ3* is partially illustrated in Figure A1 ^92^. To answer *CQ4*, we used Apache Jena Fuseki ^9^ plugin to allow running this typical type of geospatial query. A snapshot of the answer to this query is shown in Figure 6.

PREFIX rdf : *<*http://www.w3.org/rdf*>*
PREFIX owl : *<*http://www.w3.org/2002/07/owl#*>*
PREFIX rdfs : *<*http://www.w3.org/2000/01/rdf−schema#*>*
PREFIX xsd : *<*http://www.w3.org/2001/XMLSchema#*>*
PREFIX gtfs : *<*http://purl.org/net/tour−gtfs#*>*
PREFIX spatial : *<*http://jena.apache.org/spatial#*>*
SELECT * *{*
   ? busStop spatial : nearby (46.0811. 061’ miles’);
        gtfs : name?name
*}*


**Figure 7.**
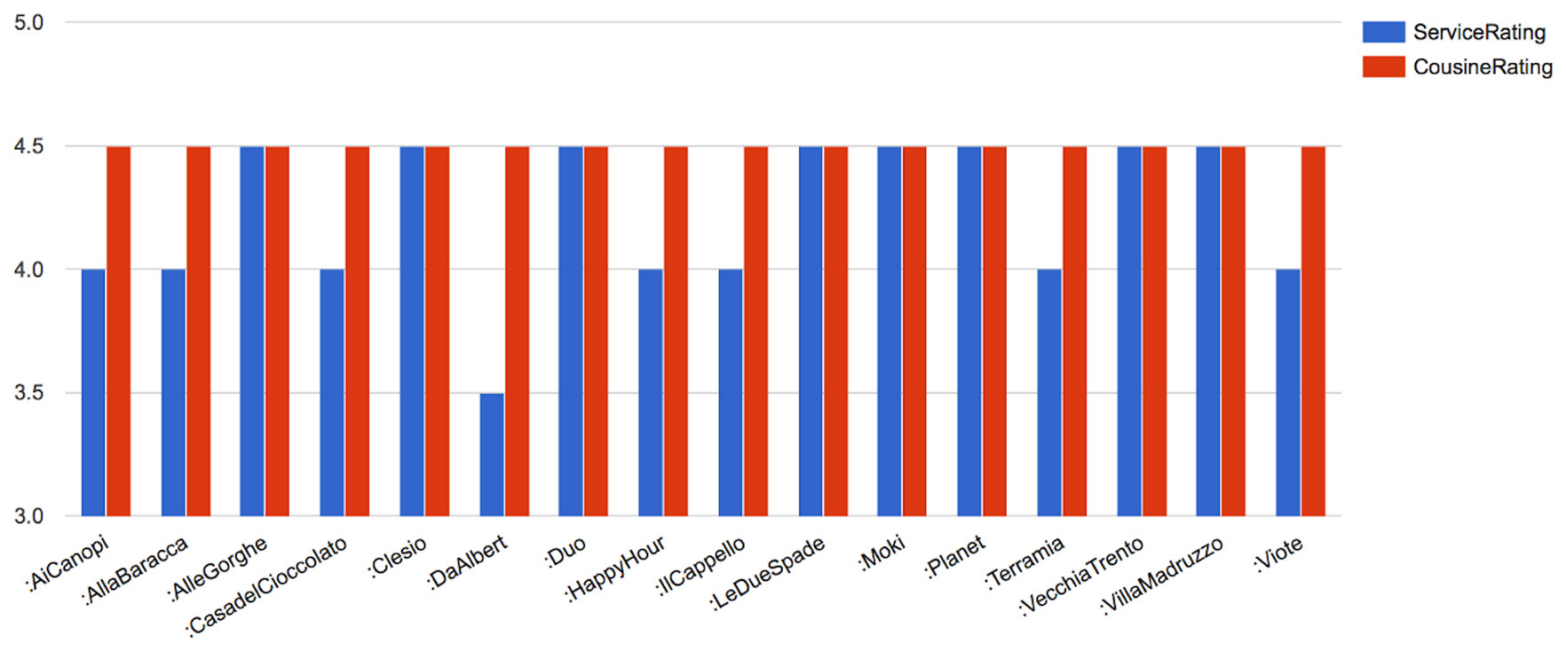
A snapshot of the SPARQL query result for quality of restaurants in Trento.

Finally, we also show, partially the results for the SPARQL query pertaining to the *CQ5* - *“List all the places where one can eat which have ratings higher than 4 stars”* in Figure 7. We omit the illustration of the rest of the CQs elucidated in Step (1) of the methodology to avoid repetition.


PREFIX rdfs : *<*http://www.w3.org/2000/01/rdf−schema#*>*
PREFIX rdf : *<*http://www.w3.org/rdf−syntax−ns#*>*
PREFIX rest : *<*http://purl.org/net/tour#*>*
SELECT  DISTINCT? eating Establishment? Service Rating? Cousine Rating
WHERE 
             *{* ? eating Establishment rdf : type? type.
? type rdfs : sub Class Of * rest:eating Estabilishment.
? eating Establishment rest : service Rating? Service Rating.
? eating Establishment rest : cousine Rating? Cousine Rating
FILTER (? Cousine Rating *>* 4).
*}*
Order  By? eating Establishment


In addition to the above twin evaluation, we also validate qualitatively as to how the proposed faceted ontology modelling methodology tackles the disentanglement of the otherwise ubiquitous problem of conceptual entanglement as stated in the introduction. To that end, the first step (Step-(1)) of the methodology concentrates on a precise documented characterization of domain analysis and competency questions identification, thereby, disentangling the complexity and multiplicity of assumptions in tourist perception (and queries). Notice that, due to the very (non-capturable) nature of perception ^93^, this step attempts reduction (and not complete elimination) of perceptual entanglement and perceptual complexity. Notice that, as briefed before, there can be successive cycles of domain analysis and competency questions enrichment/extension/abstraction in sync with successive implementational cycles of the methodology to satisfy the target coverage and informational scalability of the KG-backed tourist information service. The second step (Step-(2)) focuses on concept vocabulary determination and to that end, disentangles, the terminological entanglement otherwise prevalent in tourism ontological models. This facilitates elimination of the complexity and multiplicity of assumed meanings of terms which are used to linguistically denote concepts and properties composing the schema, e.g., *TAO* of the ontology-driven KG, e.g., *TDKG*. This step, as before, can also be repeated in successive cycles to enrich/extend/abstract the body of terms in response to increase or decreased requirements of scalability in tourist information services. The next step (Step-(2)) concentrates on the faceted hierarchy creation and sequentially tackles the rest of the levels evident in conceptual entanglement. It eliminates the complexity and multiplicity of assumptions in hierarchical entanglement by precisely modelling the subsumption hierarchy of facets and sub-concepts denoting the concepts composing the ontology-driven KG. It also eliminates the otherwise complexity and multiplicity of assumptions in intensional entanglement by precisely decorating each concept with a precise set of object properties (relating it with other concepts) and a precise set of data properties (describing it via a purpose-specific set of attribute metadata). As with the other steps, successive cycles of faceted hierarchy modelling is *key* to manage the desired level of data and informational scalability to be provided to tourists via the KG-backed information service.

Notice that, as briefed before, there can be successive cycles of domain analysis and competency questions enrichment/extension/abstraction in sync with successive implementational cycles of the methodology to satisfy the target coverage and informational scalability (e.g. with new data as in 94, 95) of the KG-backed tourist information service.

## 6 Conclusive discussion

In this paper, we proposed a faceted ontology modelling methodology for modelling, managing and analyzing diverse genres of tourism-specific domain information. The proposed methodology is theoretically and implementationally well-founded and can, to a promising extent, disentangle the conceptual entanglement inherent in state-of-the-art tourism-specific ontology-driven KGs. We also validated the proposed methodology by modelling a base version of a tourism ontology-driven KG, the results of which are promising and *extensible for reuse*. The *key* conclusive observation, from a socio-technical perspective of tourism, is that the paper highlighted a *duality* which is *ubiquitous* for tourism-specific information services, i.e., the need for acknowledging the different perceptions of different stakeholders from the same piece of target reality *as well as* the need to accommodate and harmonize such different views into a methodologically well-founded information services solution. Notice that the proposed methodology (and the baseline *TAO* and *TDKG* produced) are capable, to a large extent, to disentangle the entanglement inherent in answers provided, for instance, by the tourist information services chatbot in the motivating example (and similar information and knowledge-intensive applications).

## Figures and Tables

**Table:** 

Repository Name	Dataset description
Zenodo	Web Ontology Language (OWL) file of the DAS, S. (2024). Restaurant OWL file [Data set]. Zenodo. https://zenodo.org/doi/10.5281/zenodo.12731788 Data are available under the terms of the GNU General Public License v3.0 or later.
Zenodo	List of the SPARQL queries used to generated the answer available in text format.DAS, S. (2024). SPARQL query for eating establishment [Data set]. Zenodo. https://doi.org/10.5281/ zenodo.12799173 Data are available under the terms of the GNU General Public License v3.0 or later.
Zenodo	Snapshot of the SPARQL query result for eateries suitable for groups and that have children's menus. The table of the Image is generated using open source ontology editor Protégé ( https:// protege.stanford.edu/) DAS, S. (2024). snapshot of the SPARQL query result for eateries suitable for groups and that have children menu. Zenodo. https://doi.org/10.5281/zenodo.13120538 Data are available under the terms of the GNU General Public License v3.0 or later.

## Data Availability

No data are associated with this article.

## Declarations

- Ethics approval: Not applicable
- Consent to participate: Not applicable
- Consent for publication: Yes
- Authors’ contributions: Both authors contributed equally to this work.
