# Supplementary material for: An ontology-driven knowledge graph for tourism information management
Source: Open Res Eur. 2025 Jul 23;5:1. Originally published 2025 Jan 8. [Version 2] doi: 10.12688/openreseurope.17614.2 (PMC12338160; doi:10.12688/openreseurope.17614.2)
Supplement: Peer review history

# Reviewer report: Reviewer response for version 2

**DOI:** 10.21956/openreseurope.22224.r56994

**Published:** 2025-08-07

**Authors:** Rasha M. Ismail

**Affiliations:** 1 Information systems, Ain Shams University Faculty of Computer and Information Science, Cairo, Cairo Governorate, Egypt

Following the review of the revised article and consideration of the author’s response, the paper has been accepted for indexing.

Is the case presented with sufficient detail to be useful for teaching or other practitioners?

Partly

Is the work clearly and accurately presented and does it cite the current literature?

Partly

If applicable, is the statistical analysis and its interpretation appropriate?

Partly

Are all the source data underlying the results available to ensure full access and reuse by other researchers?

Partly

Are the conclusions drawn adequately supported by the data and analysis?

Yes

Is the background of the case’s history and progression described in sufficient detail?

Yes

Reviewer Expertise:

Data Science, Artificial Intelligence, Ontology Engineering, Information Retrieval, Software Engineering, Bioinformatics, Data Mining and Knowledge Discovery

I confirm that I have read this submission and believe that I have an appropriate level of expertise to confirm that it is of an acceptable scientific standard.

## References

[1] : An Ontology Development Methodology Based on Ontology-Driven Conceptual Modeling and Natural Language Processing: Tourism Case Study. *Big Data and Cognitive Computing* .2023;7(2) : 10.3390/bdcc7020101 10.3390/bdcc7020101

# Reviewer report: Reviewer response for version 1

**DOI:** 10.21956/openreseurope.19033.r49900

**Published:** 2025-03-06

**Authors:** Stefan Neubig

**Affiliations:** 1 Outdooractive AG, Immenstadt, Germany; 2 Technical University of Munich, ., ., Germany

First of all, I would like to thank the authors for the opportunity to review their work. The paper proposes a methodology for ontology and knowledge graph development in tourism, specifically addressing and systematically resolving conceptual entanglements based on a structured, faceted classification approach.

 While I appreciate the contribution, I do have some reservations regarding the current version of the paper, which I outline in detail below. My intention is to support the authors in further improving their work.

**General Comments**

 All in all, the paper is well written, but the sentence structure is sometimes too complex and should be simplified for the sake of readability.

**Literature Review**

- The literature review, while broad, lacks transparency (how did the authors identify the described literature?) and important details. For example, in the introduction, the authors state that the "phenomenon of conceptual entanglement" is ubiquitous in the semantic data domain; however, the authors do not mention earlier approaches to tackle this topic in the specific field of ontologies. Since this topic is the paper's primary focus, I expect a more in-depth analysis of existing approaches.
- Instead, the authors cover well-known general-purpose ontology engineering methodologies (OEMs) like METHONTOLOGY and NeOn and conclude that these methods were not instantiated to the field of tourism and/or may require tweaks. However, this ignores the fact that these methods have indeed been applied successfully and without adaption in tourism (including in my work). Moreover, the authors miss out on existing domain-specific approaches (e.g., [1, 2]).
- In their analysis of research gaps, the authors note a "major under-utilization of semantic technologies" in tourism, which I need to disagree with. A short search at Google Scholar for "tourism semantic data" uncovers a large body of literature, let alone the numerous works by Dieter Fensel (well-known in this area). Also, searching Scopus for 'TITLE-ABS-KEY ( tourism AND ( "semantic web" OR "knowledge graph" OR "ontolog*" ))' reveals more than 1200 peer-reviewed articles. What I tend to agree with, however, is the lack of a "methodological framework to harmonize different perspectives of the same target reality".
- When discussing the novelty of their approach, the authors state that the application of ontologies and KGs for tourism data is limited. But why? There are enough counterexamples, such as the works mentioned above.
- **Recommendation:** What makes the paper special is its focus on conceptual entanglements and a structured, faceted classification approach - a valuable contribution. Therefore, I believe that the authors should focus on this aspect and emphasize it even more. The literature review should be adapted accordingly; in my opinion, analyzing the use or non-use of semantic web technologies in tourism is unnecessary.

**Methodology**

- The methodology is clearly and comprehensibly demonstrated and underpinned with sufficient graphics. Only Section 3.3 (Step-2) would benefit from a little more clarity.
- What I am missing, however, is a detailed discussion of the methodology developed in the light of other existing methodologies and related literature (perhaps in a discussion section?). Competency questions and the subsequent derivation of concept hierarchies, including taxonomic and non-taxonomic relations, are generally not new. The differences should, therefore, be highlighted in much more detail.

**Evaluation**

- The ontology and the KG are evaluated based on established methods. However, the actual artifact developed in this work should instead be the proposed methodology. The evaluation carried out, although helpful, therefore misses the actual objective.
- Therefore, to effectively prove the claimed advantage of the explicit conceptual disentanglement of the developed method compared to established OEMs, an initial comparison with another (albeit inadequate) methodology would be necessary.

Is the case presented with sufficient detail to be useful for teaching or other practitioners?

No

Is the work clearly and accurately presented and does it cite the current literature?

No

If applicable, is the statistical analysis and its interpretation appropriate?

Not applicable

Are all the source data underlying the results available to ensure full access and reuse by other researchers?

Yes

Are the conclusions drawn adequately supported by the data and analysis?

No

Is the background of the case’s history and progression described in sufficient detail?

Partly

Reviewer Expertise:

Artificial Intelligence, Ontologies, Knowledge Graphs, Tourism

I confirm that I have read this submission and believe that I have an appropriate level of expertise to confirm that it is of an acceptable scientific standard, however I have significant reservations, as outlined above.

## References

[1] : Data-Driven Methodology for Knowledge Graph Generation Within the Tourism Domain. *IEEE Access* .2023;11: 10.1109/ACCESS.2023.3292153 67567-67599 10.1109/ACCESS.2023.3292153

[2] : A Knowledge Graph Perspective on Knowledge Engineering. *SN Computer Science* .2022;4(1) : 10.1007/s42979-022-01429-x 10.1007/s42979-022-01429-x

# Response

**Published:** 2025-05-23

**Authors:** SUBHASHIS DAS

**Affiliations:** Department of Computer Sciences and Automation, Universidad de Salamanca, Salamanca, Castile and León, Spain

Thank you very much for your constructive comment. Please find our explanation below.

- The literature review, while broad, lacks transparency (how did the authors identify the described literature?) and important details. For example, in the introduction, the authors state that the "phenomenon of conceptual entanglement" is ubiquitous in the semantic data domain; however, the authors do not mention earlier approaches to tackle this topic in the specific field of ontologies. Since this topic is the paper's primary focus, I expect a more in-depth analysis of existing approaches.

**ANS: **We agree that transparency in literature selection is important and appreciate the opportunity to clarify. The literature cited in Section 2 was expertly curated through a structured review of works in four intersecting domains: tourism information systems, ontology/KG modeling in tourism, general ontology engineering methodologies and knowledge organization (KO) theory. We intentionally selected works that represent foundational methodologies and recent trends, including those often cited in ontology development practice, such as METHONTOLOGY, NeOn, and Ontology Development 101. Regarding the point on "conceptual entanglement" and prior attempts to address it: we respectfully note that, as discussed in Sections 2.3–2.6, no existing methodology explicitly addresses the entire stratified structure of conceptual entanglement—from perception, to concept vocabulary, to hierarchy, to intensional properties—in a unified and methodological way. While related issues are indeed touched upon across disparate research threads (e.g., terminological heterogeneity, class hierarchy ambiguity), they are not framed or treated as an integrated conceptual modeling problem. To the best of our knowledge, this paper is the first to diagnose and formally structure the notion of conceptual entanglement in ontology-driven knowledge graph design tuned to tourism (and, therefore, the general focus of the methodology in and of itself is beyond the scope of the paper), and to propose a faceted methodology to methodically disentangle it across all levels of representation. We appreciate the reviewer’s suggestion regarding the novelty of our integrated treatment of conceptual entanglement.

- Instead, the authors cover well-known general-purpose ontology engineering methodologies (OEMs) like METHONTOLOGY and NeOn and conclude that these methods were not instantiated to the field of tourism and/or may require tweaks. However, this ignores the fact that these methods have indeed been applied successfully and without adaption in tourism (including in my work). Moreover, the authors miss out on existing domain-specific approaches (e.g., [1, 2]).

**ANS: **We thank the reviewer for pointing out the omission of key domain-specific literature, particularly the works by Chessa et al. (2023) and Simsek et al. (2022), which indeed present relevant approaches to knowledge graph construction in the tourism domain. We acknowledge this oversight and appreciate the opportunity to improve our literature coverage. At the same time, we respectfully maintain our position that existing methodologies - whether general-purpose like METHONTOLOGY or domain-specific—have not addressed the issue of conceptual entanglement in a structured and stratified way across all levels of representation (perception, vocabulary, hierarchy, intensionality). Our aim is not to dismiss existing OEMs, but to emphasize that our contribution lies in methodologically diagnosing and disentangling conceptual complexity, which we believe is an under-addressed yet foundational problem in ontology-based KG modeling. We will make this distinction clearer in the literature review by adding the suggested papers to avoid misinterpretation. We have added new text and citations to add (for the two referenced papers ) Chessa et al. (2023) propose a robust data-driven methodology for automatically generating tourism-specific knowledge graphs. Their approach leverages structured and unstructured data sources and integrates schema design, data extraction, and KG population into a single pipeline. This work exemplifies how existing methodologies can be pragmatically adapted and implemented in tourism scenarios without necessarily reengineering the conceptual modeling process. Simsek et al. (2022) offer a broad knowledge graph engineering perspective, including insights into domain-specific applications such as tourism. Their work emphasizes practical design patterns, toolchains, and knowledge reuse strategies, showing that existing frameworks can be effectively instantiated even in complex domains like tourism without extensive foundational modifications.

- In their analysis of research gaps, the authors note a "major under-utilization of semantic technologies" in tourism, which I need to disagree with. A short search at Google Scholar for "tourism semantic data" uncovers a large body of literature, let alone the numerous works by Dieter Fensel (well-known in this area). Also, searching Scopus for 'TITLE-ABS-KEY ( tourism AND ( "semantic web" OR "knowledge graph" OR "ontolog*" ))' reveals more than 1200 peer-reviewed articles. What I tend to agree with, however, is the lack of a "methodological framework to harmonize different perspectives of the same target reality".

**ANS:** The authors fully acknowledge that numerous works — including many by Dieter Fensel and others — have explored semantic technologies in tourism. To clarify: by “under-utilization,” we intended to convey not a lack of research output, but rather a limited adoption of semantically rigorous, reusable, and perceptually disentangled frameworks for modeling tourism data. As the reviewer rightly notes, there is still a gap in methodologically grounded approaches that explicitly address the problem of harmonizing divergent stakeholder perspectives within a unified ontology framework. We appreciate this point and we are glad that the reviewer aligns with our core thesis on the need for a principled framework to manage representational plurality in tourism knowledge modeling.

- When discussing the novelty of their approach, the authors state that the application of ontologies and KGs for tourism data is limited. But why? There are enough counterexamples, such as the works mentioned above.

**ANS:** The authors agree that numerous studies have applied ontologies and knowledge graphs (KGs) in the tourism domain, including notable efforts such as Harmonise, OnTour, QALL-ME, and the work by Dieter Fensel’s group. By "limited," we, however, do not refer to the number of applications, but rather to their scope and methodological grounding based multiperspective modelling of tourism data. As discussed in Section 2.6, most existing works either focus on domain-specific instantiations (e.g., recommendation systems or itinerary planners) or adopt ontological models without addressing the underlying issue of conceptual entanglement inherent in multiperspectivism, i.e., the ambiguity introduced by divergent stakeholder perceptions, inconsistent terminologies, and informal conceptual hierarchies. Our contribution lies in proposing a structured, reusable methodology and artefacts to explicitly disentangle such entanglements — across perception, vocabulary, hierarchy, and intensional properties — which is not the focus of most prior work. In this sense, our claim of novelty pertains to how ontology-driven modeling is approached rather than whether it has been applied in tourism at all.

- Recommendation: What makes the paper special is its focus on conceptual entanglements and a structured, faceted classification approach - a valuable contribution. Therefore, I believe that the authors should focus on this aspect and emphasize it even more. The literature review should be adapted accordingly; in my opinion, analyzing the use or non-use of semantic web technologies in tourism is unnecessary.

**ANS:** We are grateful for the reviewer’s appreciation of the paper’s novelty in addressing conceptual entanglement through a structured, faceted classification approach. We agree that this is a key strength of the work. However, we respectfully maintain that the discussion of the use (or lack thereof) of semantic web technologies in tourism (Section 2.1–2.2) is necessary for two reasons:

- Contextual Motivation: It highlights that most current systems lack foundational semantic structure, thereby making the case for why conceptual entanglement remains unresolved. This sets the stage for our faceted methodology as a conceptual advancement.
- Contrastive Justification: The literature on semantic web in tourism (e.g., Harmonise, QALL-ME, OnTour) often stops at technical integration or schema creation. We show (in Section 2.5–2.6) that even these efforts do not tackle conceptual entanglement — thus reinforcing our paper’s distinct contribution.

That said, we appreciate the suggestion and will consider emphasizing the faceted classification angle more strongly in any follow-up work.

Methodology

- The methodology is clearly and comprehensively demonstrated and underpinned with sufficient graphics. Only Section 3.3 (Step-2) would benefit from a little more clarity.
- What I am missing, however, is a detailed discussion of the methodology developed in the light of other existing methodologies and related literature (perhaps in a discussion section?). Competency questions and the subsequent derivation of concept hierarchies, including taxonomic and non-taxonomic relations, are generally not new. The differences should, therefore, be highlighted in much more detail.

**ANS:** We thank the reviewer for their positive remarks on the clarity and completeness of the methodology and graphics. Regarding the comment on methodological novelty, we respectfully point out that our manuscript does include a dedicated section (Section 2.3 and 2.4) that reviews existing ontology and knowledge graph development methodologies, including prominent approaches such as METHONTOLOGY, NeOn, and Ontology Development 101. Furthermore, in Section 2.6 (“Novelty”), we explicitly articulate how our methodology differ - particularly in addressing the stratified nature of conceptual entanglement (from perception to intensionality), which, to our knowledge, has not been comprehensively treated in existing methodologies (even when tuned for tourism). While competency questions and hierarchical modeling are indeed well-established components in ontology design, our key contribution lies in systematically aligning each step with the disentanglement of conceptual ambiguity across multiple levels, grounded in principles from faceted knowledge organization and applied ontology. We acknowledge that the distinction may not stand out clearly to readers unfamiliar with KO theory, and we refer the following publications regarding the details of the methodology in and of itself:

- Bagchi M, Das S: Conceptually disentangled classificatory ontologies. In: 15th Seminar on Ontology Research in Brazil (ONTOBRAS), Brazil, 2022.
- Bagchi M, Das S: Disentangling domain ontologies. In: 19th IRCDL (The Conference On Information And Research Science Connecting to Digital and Library Science), Bari, Italy, 2023.

Evaluation

- The ontology and the KG are evaluated based on established methods. However, the actual artifact developed in this work should instead be the proposed methodology. The evaluation carried out, although helpful, therefore misses the actual objective.
- Therefore, to effectively prove the claimed advantage of the explicit conceptual disentanglement of the developed method compared to established OEMs, an initial comparison with another (albeit inadequate) methodology would be necessary.

**ANS:** The combined contribution of this work is the methodology for faceted ontology modeling and conceptual disentanglement and the resulting semantic artifacts (TAO and TDKG). Our choice to evaluate the resulting ontology and knowledge graph using established qualitative and quantitative methods (Section 5) was intended as a proxy for validating the methodology itself, by showing that it yields structured, reusable, and query-competent outputs from complex, perceptually diverse domains. We fully agree that a direct methodological comparison with other ontology engineering methodologies (OEMs) would further strengthen the case for our disentanglement approach. However, such a comparative study would require extending the current work into a broader empirical or meta-ontological benchmarking framework, which we view as an important next step rather than part of the current scope of this paper. That said, we do highlight (in Sections 2.3 and 2.6) the conceptual limits of existing OEMs in addressing entanglement at multiple representational levels. The authors hope that it positions the proposed methodology as a theoretically motivated and practically viable contribution toward bridging that gap.

# Reviewer report: Reviewer response for version 1

**DOI:** 10.21956/openreseurope.19033.r50249

**Published:** 2025-02-07

**Authors:** Rasha M. Ismail

**Affiliations:** 1 Information systems, Ain Shams University Faculty of Computer and Information Science, Cairo, Cairo Governorate, Egypt

The paper "An ontology-driven knowledge graph for tourism information management" offers a solution to address the complexity of modern tourism-related queries through an AI-driven ontology-based knowledge graph.

- The paper addresses a highly relevant problem in the tourism industry by suggesting an innovative use of AI and ontology-driven knowledge graphs. The methodology is well-founded, and the paper provides a good example of its implementation with a populated knowledge graph.
- While the paper demonstrates the use of the knowledge graph with 4264 RDF statements, it would be useful to explore how these graphs can be continuously updated and managed in real-time, especially in dynamic environments like tourism.
- The author need to give more detail on how the proposed system performs in practice, such as user experience, accuracy of the AI’s responses, and comparisons with existing systems.
- The paper claims to validate the proposed methodology by developing a knowledge graph, but it does not provide a rigorous empirical evaluation or real-world testing with actual users. Without user feedback or performance benchmarks, it is unclear how effective the proposed solution is in practical tourism applications.
- The paper does not discuss strategies for maintaining data quality, handling inconsistencies, or updating the graph in response to new tourism information.
- The paper does not compare its proposed approach to existing tourism ICT solutions, such as recommendation systems, chatbots, or other AI-driven knowledge representation techniques.
- Some sentences are long and repetitive, so please simplify these sentences and Some minor grammatical errors are needed to be reviewed.

Is the case presented with sufficient detail to be useful for teaching or other practitioners?

Partly

Is the work clearly and accurately presented and does it cite the current literature?

Partly

If applicable, is the statistical analysis and its interpretation appropriate?

Partly

Are all the source data underlying the results available to ensure full access and reuse by other researchers?

Partly

Are the conclusions drawn adequately supported by the data and analysis?

Yes

Is the background of the case’s history and progression described in sufficient detail?

Yes

Reviewer Expertise:

Data Science, Artificial Intelligence, Ontology Engineering, Information Retrieval, Software Engineering, Bioinformatics, Data Mining and Knowledge Discovery

I confirm that I have read this submission and believe that I have an appropriate level of expertise to confirm that it is of an acceptable scientific standard, however I have significant reservations, as outlined above.

## References

[1] : An Ontology Development Methodology Based on Ontology-Driven Conceptual Modeling and Natural Language Processing: Tourism Case Study. *Big Data and Cognitive Computing* .2023;7(2) : 10.3390/bdcc7020101 10.3390/bdcc7020101

# Response

**Published:** 2025-05-23

**Authors:** SUBHASHIS DAS

**Affiliations:** Department of Computer Sciences and Automation, Universidad de Salamanca, Salamanca, Castile and León, Spain

Thank you very much for your constructive comment. Please find our explanation below.

- The paper addresses a highly relevant problem in the tourism industry by suggesting an innovative use of AI and ontology-driven knowledge graphs. The methodology is well-founded, and the paper provides a good example of its implementation with a populated knowledge graph.

ANS: Thank you. We appreciate the reviewer’s thoughtful observation on our work.

- While the paper demonstrates the use of the knowledge graph with 4264 RDF statements, it would be useful to explore how these graphs can be continuously updated and managed in real-time, especially in dynamic environments like tourism.
- The paper does not discuss strategies for maintaining data quality, handling inconsistencies, or updating the graph in response to new tourism information.

ANS: for both points together: The esteemed reviewer’s concern regarding the continuous update, real-time management, and data quality maintenance of the ontology-driven knowledge graph (KG) is highly pertinent, especially in dynamic domains such as tourism. While our current work focuses on the foundational methodology and an initial instantiation of the Tourism Applications Ontology (TAO) and Tourism Domain Knowledge Graph (TDKG), we fully acknowledge the importance of addressing these lifecycle management concerns.   We would like to respectfully highlight that the modular and faceted design of our ontology, as described in Sections 3.4 and 3.5 was specifically chosen to support future extensibility and evolution of the KG. This is also briefly touched upon in the concluding discussion (Section 6), where we note that our methodology can be “repeated again and again in a cyclic fashion,” precisely to accommodate ongoing updates and maintenance.   While real-time updates, inconsistency resolution, and dynamic synchronization mechanisms are indeed essential next steps, they fall beyond the immediate scope of the current study. We consider this a valuable direction for future research building on the reusable infrastructure proposed here.

- The author need to give more details on how the proposed system performs in practice, such as user experience, accuracy of the AI’s responses, and comparisons with existing systems.

ANS: The observation regarding practical evaluation aspects such as user experience, response accuracy, and benchmarking against existing systems is insightful. We agree that such dimensions are critical for a full-scale deployment. However, we would like to clarify that the current work is primarily methodological and conceptual in nature. The focus of the paper is on advancing a well-founded faceted ontology modeling methodology, exemplified through the construction and population of a tourism-specific Knowledge Graph (TDKG). As noted in Sections 4 and 5, we validate the model by executing a series of representative SPARQL queries derived from real-world competency questions (CQs), which serve as a proxy for assessing the responsiveness and expressivity of the system. We acknowledge that detailed user experience studies and empirical comparisons with existing tourism information systems are important, but they are intentionally out of the scope of this first methodology-driven ontology and KG paper. We believe this work lays the groundwork for such evaluations in subsequent studies and systems-level implementations.

- The paper claims to validate the proposed methodology by developing a knowledge graph, but it does not provide a rigorous empirical evaluation or real-world testing with actual users. Without user feedback or performance benchmarks, it is unclear how effective the proposed solution is in practical tourism applications.

ANS: The methodology’s novelty lies in its stratified approach to disentanglement (perception, terminology, hierarchy, intensionality), which is not directly comparable to monolithic ontology-building techniques. To that end, the paper emphasizes methodological validation and evaluation (e.g., adherence to OntoClean principles, SPARQL query results) over competitive benchmarking, as the latter requires a shared dataset and task framework and is beyond this study’s scope. The evaluation further aligns with Gomez-Perez’s guidelines for ontology assessment, prioritizing correctness and fitness-for-purpose over comparative metrics. All in all, an in-depth control variable analysis is beyond the scope of this work.

- The paper does not compare its proposed approach to existing tourism ICT solutions, such as recommendation systems, chatbots, or other AI-driven knowledge representation techniques.

ANS: We appreciate the reviewer’s suggestion to include comparative analysis with existing tourism ICT solutions, such as recommendation systems and chatbots. Our intent in this paper, however, is not to propose an end-user system like a chatbot or a recommender but rather to present a generalizable and modular ontology-driven knowledge graph modeling methodology which could serve as the semantic backbone for such systems. As described in Section 2.2 and Section 2.3 of the literature review, we have provided an extensive overview of existing ontology-based tourism systems and methodologies (often tuned to tourism). We also differentiate our contribution by emphasizing the issue of conceptual entanglement and by proposing a faceted, step-by-step methodology for its systematic disentanglement - an aspect largely overlooked in current systems as noted in Section 2.6. Therefore, rather than a direct performance comparison with specific ICT tools, our contribution lies in addressing foundational limitations in schema and knowledge base design, which are often assumed as given in other tourism applications. Given the above, we fully agree that integrating our model into existing ICT systems for benchmarking purposes is an important avenue for future work, building on the ontological infrastructure developed here.

- Some sentences are long and repetitive, so please simplify these sentences and Some minor grammatical errors are needed to be reviewed.

ANS: Thank you. We’ve adjusted as much as possible following the limitations of the paper argumentation.

# Reviewer report: Reviewer response for version 1

**DOI:** 10.21956/openreseurope.19033.r50241

**Published:** 2025-02-05

**Authors:** Hongyin Zhu

**Affiliations:** 1 360 search department, Qihoo 360, Beijing, China

Pros:

 This paper proposes a novel perspective, considering the semantic knowledge construction of multi-party interest scenarios. Since the personalized needs of user groups are taken into account when querying, the implementation is more complex and challenging. I think the author has thought deeply about the tourism knowledge graph component, which is very valuable for solving the ambiguity of large language model technology.

 Cons:

 1. "Lao Tzu once famously said, “a journey of a thousand miles begins

 with a single step.” The summary in the Abstract is very interesting. How do you think this wise quote relates to the tourism KG?

 2. "be exploited by all players in 

 tourism - tourists, businesses, governments, human and artificial agents, researchers, analysts, etc." How to determine the concept set of all parties?

 3. What are the advantages and disadvantages of tourism AI services based on knowledge graphs and direct replies based on chatgpt? How do you think fine-grained knowledge graph modeling and coarse-grained knowledge graph modeling are balanced in tourism AI?

 4. Aspect-oriented ontology modeling is a novel method in tourism knowledge graphs. The tourism knowledge graph constructed in this paper makes full use of structured data modeling. Are the unstructured and semi-structured data in the tourism field mentioned in [1][2] helpful to this paper?

 [1] Xie, Jiawang, et al., 2021 [Ref-1].

 [2] Zhu, Hongyin, et al.,2023 [Ref-2]

 5. How do you define the scope of concepts in the ontology you built, and on what principles?

 6. How are the ontology construction and instance-level modeling aligned? How complete are the instance attributes? What is the number of entities and relations?

 7. The concept entanglement problem in the tourism field is a challenging problem. Why are existing methods difficult to solve? Integrating the needs of multiple stakeholders is a challenging problem. What is the difficulty? How to solve it?

 8. The author mentioned many novel concepts and understandings, such as medical tourism, Osteria, Bar, Bistro, Alpine Hut. Is it necessary to make the ontology of food and beverage [3] more refined?

 [3] Zhu, H.,2022 [Ref-3].

 9. The subsection Critical analysis of research gaps is very well written and has a novel perspective. It is recommended to put it in the main text instead of the literature review.

 10. This paper analyzes user queries in more detail. Does it support the conversion of natural language queries to SPARQL query language?

 11. Does the tourism AI system have an accessible website? What are your insights on spatiotemporal modeling and event modeling in tourism knowledge graphs?

 12. The Evaluation section lacks a clear subsection hierarchy and quantitative experimental comparisons, including comparisons with other works and in-depth control variable analysis.

Is the case presented with sufficient detail to be useful for teaching or other practitioners?

Yes

Is the work clearly and accurately presented and does it cite the current literature?

Yes

If applicable, is the statistical analysis and its interpretation appropriate?

Partly

Are all the source data underlying the results available to ensure full access and reuse by other researchers?

Partly

Are the conclusions drawn adequately supported by the data and analysis?

Yes

Is the background of the case’s history and progression described in sufficient detail?

Yes

Reviewer Expertise:

Knowlege graph, natural langauge processing

I confirm that I have read this submission and believe that I have an appropriate level of expertise to confirm that it is of an acceptable scientific standard, however I have significant reservations, as outlined above.

## References

[1] : Construction of Multimodal Chinese Tourism Knowledge Graph. *Springer Singapore* .2021; 10.1007/978-981-16-5943-0_2

[2] : Pre-training language model incorporating domain-specific heterogeneous knowledge into a unified representation. *Expert Systems with Applications* .2023;215: 10.1016/j.eswa.2022.119369 10.1016/j.eswa.2022.119369

[3] : MetaAID: A Flexible Framework for Developing Metaverse Applications via AI Technology and Human Editing. *arXiv* .2022; Reference source

# Response

**Published:** 2025-05-23

**Authors:** SUBHASHIS DAS

**Affiliations:** Department of Computer Sciences and Automation, Universidad de Salamanca, Salamanca, Castile and León, Spain

Thank you very much for your constructive comment.

 Please find our explanation below.

 1. "Lao Tzu once famously said, “a journey of a thousand miles begins with a single step.” The summary in the Abstract is very interesting. How do you think this wise quote relates to the tourism KG? ANS: The quote by Lao Tzu, while bearing rich socio-literary wisdom, is also central as a general message and motivation to the technological approach proposed in the paper. The modern ecosystem of tourism is extremely multi-faceted and multi-context dependent, so much so that tourists, planning on an excursion ( *“a journey of a thousand miles”*), have to make the first step ( *“a single step”*) of gathering information relative to an entire range of queries from as basic as “Where to eat?” to as complex as “List all the eating establishments which are suitable for groups and also provide children menu”. Such a list becomes even more complicated due to the intermixing of different perceptions, languages, cultures and economies in which tourists are enmeshed in. This is the motivational essence that the authors have tried to capture bidirectionally between the quote and the motivation of the work behind the tourism KG.

 2.  "be exploited by all players in  tourism - tourists, businesses, governments, human and artificial agents, researchers, analysts, etc." How to determine the concept set of all parties? ANS: This is an important question. In fact, no single ontology-driven KG can model and represent the concept set of all parties. That is the very basis of the methodological framework of conceptual disentanglement underlying the modelling of the TAO and TDKG, i.e., the fact the perception of a domain of discourse (here, tourism) is specific to the community of practice (“parties”) who are interested in modelling and instantiating the domain and the concept set for that community of practice emerges from that specific perception. Therefore, while encoding some overlapping concepts, the different parties, e.g., tourists, researchers, governments, etc., have an overall different concept set specific to their perception.

 3. What are the advantages and disadvantages of tourism AI services based on knowledge graphs and direct replies based on chatgpt? How do you think fine-grained knowledge graph modeling and coarse-grained knowledge graph modeling are balanced in tourism AI? ANS: The reviewer raised an important perspective regarding the comparative positioning of knowledge graph-based tourism AI services versus large language model-based systems, such as ChatGPT. As clarified in our manuscript, the core contribution of our work lies in proposing a theoretically grounded methodology for ontology-driven knowledge graph (KG) construction - particularly focused on handling the multi-layered conceptual entanglement inherent in tourism information modeling. While we acknowledge the growing prominence of open-domain conversational agents, our work deliberately centers on building high-quality, semantically explainable, and reusable domain-specific KGs. Such structured KGs, as evidenced through the competency question-driven evaluation in Section 5, enable fine-grained, trustworthy, and context-aware responses - especially suited to tourism use cases involving complex filters (e.g., amenities, accessibility, suitability, events). Unlike purely generative systems, our model ensures transparency and ontological clarity, which are often critical for decision-making in tourism contexts. On the modeling granularity, the faceted ontology approach adopted in our methodology allows natural balancing between fine and coarse grained modeling. By structuring information hierarchically (as shown in Section 3.4), we support both detailed queries (e.g., children menus in specific suburbs) and abstract reasoning (e.g., types of eating establishments). This enables scalable reuse without overfitting to one level of granularity, in line with our aim to build an expandable and interoperable tourism information infrastructure.

 4. Aspect-oriented ontology modeling is a novel method in tourism knowledge graphs. The tourism knowledge graph constructed in this paper makes full use of structured data modeling. Are the unstructured and semi-structured data in the tourism field mentioned in [1][2] helpful to this paper? ANS: Thank you very much for bringing our attention to the (un/semi)structured data as referenced to in the cited literature. They would be essential for the next phase of developing TAO and TDKG and we’ll cite them to that effect in the paper. (Added new text and citation in the papers in section -5 as below :-) ) Notice that, as briefed before, there can be successive cycles of domain analysis and competency questions enrichment/extension/abstraction in sync with successive implementational cycles of the methodology to satisfy the target coverage and informational scalability (e.g., with new data as in [1.2]) of the KG-backed tourist information service.

 5. How do you define the scope of concepts in the ontology you built, and on what principles?

 6. How are the ontology construction and instance-level modeling aligned? How complete are the instance attributes? What is the number of entities and relations? ANS: combined for 5 and 6: The scope of concepts in our ontology is defined through a systematic methodology grounded in domain and competency question (CQ) analysis (Section 3.2). This ensures alignment between the ontology and real-world information needs in tourism. The conceptual model is shaped using faceted classification principles (Section 3.4), which allow for organizing concepts hierarchically and systematically, reducing ambiguity and enabling reusability. We deliberately adopted WordNet, schema.org, and other authoritative sources (Section 3.3) to standardize the vocabulary and ensure semantic clarity. The ontology construction and instance-level modeling are tightly coupled through our four-step faceted ontology modeling methodology (Section 3), culminating in a structured OWL implementation (Section 4). The Tourism Applications Ontology (TAO) provides the schema, and the Tourism Domain Knowledge Graph (TDKG) instantiates it using real-world data.   Each competency question in Step 1 maps to concrete class-attribute-property combinations, ensuring that instance-level data are both semantically justified and structurally compliant with the ontology. This alignment was verified using OWL reasoners (e.g., HermiT and Fact++). The attributes of instances in the TDKG were carefully curated to reflect the full spectrum of information required to answer the identified competency questions (Section 3.2 and 5). The attributes span amenities, contact details, spatial and temporal metadata, menu options, and ratings (Section 3.4.2), with data types rigorously defined (e.g., boolean, string, float, dateTime) in the OWL implementation (Section 4). Completeness was assessed through SPARQL-based evaluations (Figures 4 - 7), demonstrating that all CQs could be operationalized using available data.

 7. The concept entanglement problem in the tourism field is a challenging problem. Why are existing methods difficult to solve? Integrating the needs of multiple stakeholders is a challenging problem. What is the difficulty? How to solve it? ANS: Thank you for raising very interesting points. Existing methods often struggle with concept entanglement because they assume a fixed perspective, whereas tourism involves multiple overlapping perspectives via; perceptions, terminologies, and hierarchies. This leads to ambiguity and poor reusability (Section 2.5). Integrating stakeholder needs is difficult due to diverse, sometimes conflicting viewpoints across tourists, providers, and agencies. Without a structured way to separate and reconcile these, models become inconsistent. Our methodology addresses this via faceted ontology modeling (Section 3), which disentangles complexity across four levels — perception, terminology, hierarchy, and intensionality — and allows modular incorporation of stakeholder views. This ensures clarity, scalability, and interoperability (Sections 3.5 and 6).

 8. The author mentioned many novel concepts and understandings, such as medical tourism, Osteria, Bar, Bistro, Alpine Hut. Is it necessary to make the ontology of food and beverage [3] more refined? [3] Zhu, H.,2022 [Ref-3]. ANS: The focus of our ontology, as described in Section 3.1 and elaborated in Steps 1 - 3 (Sections 3.2–3.4), is tourism-specific information services and not food and beverage categorization *per se*. Concepts such as Osteria, Bistro, and Alpine Hut are included only insofar as they contribute to modeling eating establishments as part of the tourist experience in Trentino. We acknowledge that specialized ontologies like [3] may offer deeper and valuable refinements in food and beverage classification for future adaptations. However, for this specific paper, our goal is not to replicate or extend such vertical domain ontologies, but to ensure interoperability and reusability by incorporating selected elements (e.g., from schema.org/FoodEstablishment, BBC ontology) relevant to tourist decision-making (see Section 3.1 and 3.3). Thus, while further refinement in food/beverage subtypes could be pursued in future domain extensions, we believe the current level of granularity is both sufficient and appropriate for the intended scope of tourism knowledge representation, without compromising conceptual clarity or usability. Q. The subsection Critical analysis of research gaps is very well written and has a novel perspective. It is recommended to put it in the main text instead of the literature review. ANS: Thank you. We’ve adjusted as much as possible following the limitations of space and length. Q. This paper analyzes user queries in more detail. Does it support the conversion of natural language queries to SPARQL query language? ANS: The conversion of natural language queries to SPARQL query language is extrinsic to the scope and theme of the paper. However, the authors encourage the readers, following the comment of the esteemed reviewer, to try out any of the free and open source NL to SPARQL query rewriters  for purposes of the ontology/KG requirements satisfaction and evaluation. Q. . Does the tourism AI system have an accessible website? What are your insights on spatiotemporal modeling and event modeling in tourism knowledge graphs? ANS: The paper proposed an ontology and an ontology-driven KG for the tourism domain and not a full-fledged AI system with a website (which, however, can have the KG as one of its underlying knowledge base). While the authors discuss the potential for the ontology-driven knowledge graph to underpin applications like a tourism information services chatbot, and validate their methodology by developing a populated knowledge graph (TDKG), there is no indication that this system is available as a user-facing website, a proposal of the full fledged system with website is beyond the scope of the paper. The following points are to be noted regarding insights on spatiotemporal modelling and event modelling in tourism knowledge graphs: The paper details how some of these aspects are modelled within the Tourism Applications Ontology (TAO) and its populated knowledge graph, the Tourism Domain Knowledge Graph (TDKG). Event Modelling:

- The ontology includes an `Event` class, drawing inspiration from the DOLCE top-level ontology.
- Events can be specialised into subclasses based on their theme or the people they are for, such as children's events, cultural events, seminars, or social events.
- Examples of themes mentioned are music or food.
-  Events are associated with temporal attributes, including duration (in days), start date-time, and end date-time.
- Events are linked to the buildings where they are hosted via the symmetrical relations hosts and isHostedBy.
- The knowledge graph supports queries related to events, such as asking which events will be scheduled at a specific eating establishment in the future, or where and when a known event will be held.

Spatiotemporal Modelling:

- The ontology models spatial information through the Administrative Division class, which includes Country, City, and Suburb.
- These administrative divisions are interrelated using the part-of relation, for example, showing that a Suburb is part of a City, and a City is part of a Country.
- Buildings, including Eating Establishments, are linked to their geographical locations (country, city, suburb) using relations like `addressCountry`, `addressCity`, and `addressSuburb`.
- Specific spatial attributes like `address street`, `house number`, and `postcode` are defined for buildings.
- The developed knowledge graph supports geospatial queries such as finding the nearest eating establishments surrounding a transportation stop. This type of query requires modelling and leveraging spatial relationships between different entities in the graph.
- The paper also notes an Event-centric Tourism Knowledge Graph (ETKG) capable of modelling both temporal and spatial dynamics of tourist trips. This reinforces the relevance of these modelling aspects in the tourism domain.

The methodology's step-by-step approach, including Competency Question identification and Faceted Hierarchy Creation, is designed to capture the necessary concepts, attributes, and relations to support complex queries involving both spatial and temporal information related to tourism entities like eating establishments and events. The resulting TAO and TDKG are structured to provide fine-grained information services by interrelating concepts spatially and temporally where relevant. Q. The Evaluation section lacks a clear subsection hierarchy and quantitative experimental comparisons, including comparisons with other works and in-depth control variable analysis. ANS: The methodology’s novelty lies in its stratified approach to disentanglement (perception, terminology, hierarchy, intensionality), which is not directly comparable to monolithic ontology-building techniques. To that end, the paper emphasizes methodological validation (e.g., adherence to OntoClean principles, SPARQL query results) over competitive benchmarking, as the latter requires a shared dataset and task framework and is beyond this study’s scope. The evaluation further aligns with Gomez-Perez’s guidelines for ontology assessment, prioritizing correctness and fitness-for-purpose over comparative metrics. All in all, an in-depth control variable analysis is beyond the scope of this work.
